# Spinal cord repair is modulated by the neurogenic factor Hb-egf under direction of a regeneration-associated enhancer

**DOI:** 10.1038/s41467-023-40486-5

**Published:** 2023-08-11

**Authors:** Valentina Cigliola, Adam Shoffner, Nutishia Lee, Jianhong Ou, Trevor J. Gonzalez, Jiaul Hoque, Clayton J. Becker, Yanchao Han, Grace Shen, Timothy D. Faw, Muhammad M. Abd-El-Barr, Shyni Varghese, Aravind Asokan, Kenneth D. Poss

**Affiliations:** 1 Duke Regeneration Center, Duke University, Durham, NC USA; 2 Department of Cell Biology, Duke University Medical Center, Durham, NC USA; 3 Université Côte d’Azur, Inserm, CNRS, Institut de Biologie Valrose, Nice, France; 4 Department of Surgery, Duke University Medical Center, Durham, NC USA; 5 Department of Molecular Genetics and Microbiology, Duke University School of Medicine, Durham, NC USA; 6 Department of Orthopedic Surgery, Duke University School of Medicine, Durham, NC USA; 7 Department of Cardiovascular Surgery of the First Affiliated Hospital and Institute for Cardiovascular Science, Soochow University, Suzhou, Jiangsu China; 8 Duke Institute for Brain Sciences, Duke University, Durham, NC USA; 9 Department of Neurosurgery, Duke University Medical Center, Durham, NC USA; 10 Department of Mechanical Engineering and Materials Science, Duke University, Durham, NC USA; 11 Department of Biomedical Engineering, Duke University, Durham, NC USA

**Keywords:** Regeneration, Regeneration, Growth factor signalling, Spinal cord injury

## Abstract

Unlike adult mammals, zebrafish regenerate spinal cord tissue and recover locomotor ability after a paralyzing injury. Here, we find that ependymal cells in zebrafish spinal cords produce the neurogenic factor Hb-egfa upon transection injury. Animals with *hb-egfa* mutations display defective swim capacity, axon crossing, and tissue bridging after spinal cord transection, associated with disrupted indicators of neuron production. Local recombinant human HB-EGF delivery alters ependymal cell cycling and tissue bridging, enhancing functional regeneration. Epigenetic profiling reveals a tissue regeneration enhancer element (TREE) linked to *hb-egfa* that directs gene expression in spinal cord injuries. Systemically delivered recombinant AAVs containing this zebrafish TREE target gene expression to crush injuries of neonatal, but not adult, murine spinal cords. Moreover, enhancer-based HB-EGF delivery by AAV administration improves axon densities after crush injury in neonatal cords. Our results identify Hb-egf as a neurogenic factor necessary for innate spinal cord regeneration and suggest strategies to improve spinal cord repair in mammals.

## Abstract (web summary)

Zebrafish can regenerate after paralyzing spine injuries and regain locomotor ability, unlike mammals. Here authors show that the neurogenic factor Hb-egf promotes spinal cord regeneration in zebrafish and is regulated by an enhancer that can similarly direct expression in the pro-regenerative setting of neonatal mice.

## Introduction

Adult mammals including humans display little or no regeneration in response to spinal cord injury, leading to sensorimotor deficits and paralysis. The pathophysiology of spinal cord injury is multifaceted and complex. It requires treatments acting not only on movement and sensation, but also on associated complications of respiratory, cardiovascular and urinary systems^1^. Rehabilitation can minimize deficits after moderate spinal cord injuries, and electrical stimulation has been shown to provide some benefits in recent years. Yet, they both fail to fully restore cell composition and function of the endogenous, damaged tissue, leaving individuals with spinal cord injury paralyzed for life in most cases. Consistent with initial results in opossum^2^, a recent study reported that the neonatal mouse spinal cord can mount a regenerative response to spinal cord injury that permits the growth of long projecting axons through lesion sites^3^. Stage-specific roles of immune cells like microglia were implicated as one component of this transient regenerative capacity^3^. Other candidate stage-dependent influences on the potential for mammalian spinal cord regeneration include the tissue composition of scar-forming fibroblasts or glial cells^4–8^, developmental changes in growth, survival, or metabolic programs^9–11^, and alterations in chromatin environment or transcription factor binding that might affect gene expression underlying any of the above processes^12,13^.

The study of high regenerative capacity in non-mammalian vertebrates is a centuries-old strategy to identify determinants of regeneration. Of note, laboratory zebrafish can fully regenerate and recover function after complete spinal cord transection and crush injuries, at any point in life^14–16^. Spinal cord injury in adult zebrafish triggers proliferation of ependymo-radial glial cells (ERGs) lining the central canal, also referred to as “radial glia”, “tanycytes” and “ependymoglia”^15–21^. A subpopulation of glial progeny helps form a tissue bridge between the rostral and caudal stumps, events that depends on signaling by Fibroblast growth factors (Fgfs) and Connective tissue growth factor a (Ctgfa)^19,22^. Though a topic of debate, these bridges have been proposed to serve as possible scaffolds across which axons extend to re-establish functional connections^19,22^. A variety of intrinsic and extracellular factors have been implicated in guidance of axons across the lesion site in adult zebrafish, among which are secreted proteins (Sema4D), extracellular matrix components (Tenascin-C), scaffolding proteins (Syntenin-A), transcription factors (Atf3 and 6), microRNAs (mi133) and immune-cell derived factors (Neurotrophin 3)^23–28^.

ERGs are also the source of new neurons in the vicinity of the lesion^16,17^. Although zebrafish ERGs are grossly homogeneous in morphology, their dorsoventral position with respect to the central canal has been reported to define their neuronal progeny after spinal cord injury, predicting whether they give rise to motoneurons or interneurons^15,20,29^. Similar progenitors with the ability to produce neurons also exist in the mammalian spinal cord, but they possess low neurogenic potential in vivo^30^. In this study, we examined transcriptomes for factors that are induced during the regeneration of injured adult zebrafish spinal cords and that may have neurogenic roles, and our experiments highlighted a key role for signaling by Heparin-binding epidermal growth factor (Hb-egf). Hb-egf is a secreted glycoprotein member of the epidermal growth factor (Egf) family of growth factors, and it signals through Egf receptor (Egfr) and the related receptor tyrosine kinase ErbB4^31^. Binding of Hb-egf to its receptors triggers known downstream cascades, the most common of which are the mitogen-activated protein kinase (MAPK)/extracellular signal-regulated kinase (ERK) pathway, the (PIK3)/protein kinase B (Akt) pathway and the phospholipase Cγ (PLCγ) pathway. Wnt and Notch signaling cascades also act downstream of Hb-egf. Hb-egf has been implicated in neurogenesis during retinal regeneration in zebrafish using morpholino knockdown techniques^32^, and it has also been reported to induce neurogenesis in the hippocampal subventricular zone and dentate gyrus in mice and rats^33,34^.

Here, we employed a variety of molecular genetic, biomaterials, and profiling approaches in zebrafish to demonstrate a new role for Hb-egfa, produced by one of the zebrafish *hb-egf* paralogs, in innate spinal cord regeneration. We find that Hb-egfa is synthesized in ERGs at lesion sites and is required for neurogenesis and, ultimately, for axon crossing events and functional recovery. With a cross-species strategy that enlists *hb-egfa*-linked DNA control elements of zebrafish, we find that targeted HB-EGF delivery can boost axon density in mammalian spinal cord injury sites.

## Results

### Spinal cord injury induces expression of *hb-egf* genes

**Fig. 1:**
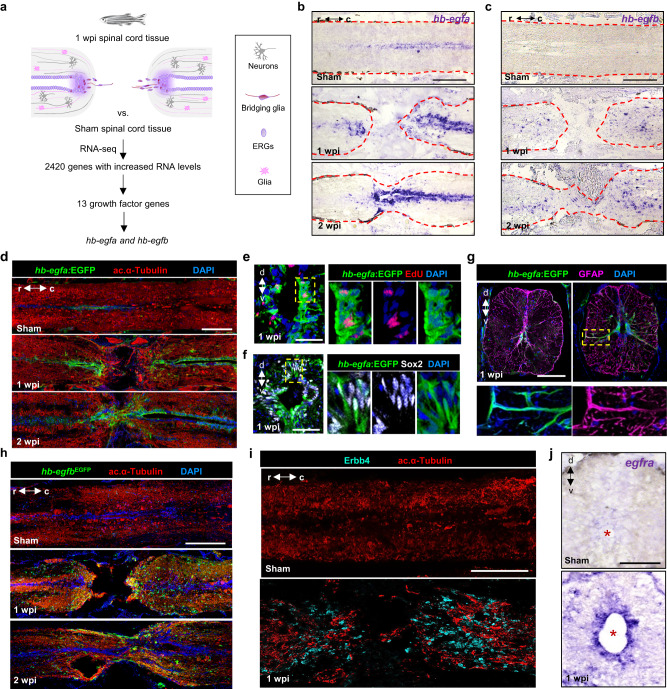
*hb-egf* genes are induced in zebrafish spinal cord after injury. **a** Strategy used to identify regulators of spinal cord regeneration. **b**, **c** In situ hybridization for *hb-egfa and hb-egfb* on longitudinal sections of zebrafish spinal cord at 1 and 2 wpi, and in sham-injured controls. Dashed lines delineate spinal cord. **d** Sections of spinal cord tissue indicating *hb-egfa:EGFP* BAC reporter expression (green) in sham, 1 and 2 wpi animals. Acetylated α-Tubulin (red) stains axons. **e** EdU (red) incorporation assay for cell cycling at 1 wpi in transverse sections of *hb-egfa:EGFP* spinal cord. **f** Assays for Sox2 (white) expression at 1 wpi in transverse sections of *hb-egfa:EGFP* spinal cord. **g** Cross-section of *hb-egfa:EGFP* spinal cord at 1 wpi showing *hb-egfa* expression in ependymoradial-glial cells. GFAP (magenta) stains glial cells. Dashed box is magnified at bottom. **h** Expression of *hb-egfb*^*EGFP*^ in sham injured and injured adult spinal cords at 1 and 2 wpi. **i** Longitudinal sections of spinal cord indicating expression of Erbb4 receptor (cyan) after sham injury or at 1 wpi. **j** Transverse sections of adult spinal cord showing expression of *egfra* mRNA by in situ hybridization after sham injury or at 1 and 2 wpi. *N* = 3 in (**b**–**d**) and (**g**–**j**); *N* = 2 in (**e**, **f**). Asterisks in (**j**) indicate central canal. Dashed area in (**e**, **f**) indicate regions magnified. Scale bars 200 μm in (**b**–**d**, **h**, **i**); 100 μm in (**g**, **j**); 50 μm in (**e**, **f**). d dorsal, v ventral, r rostral, c caudal.

In an expression profiling experiment for potentially neurogenic growth factor genes induced during zebrafish spinal cord regeneration, we noted that both gene paralogs of *heparin-binding epidermal growth factor*, *hb-egfa* and *hb-egfb*, display sharp increases in RNA levels at 1 week after transection injury (wpi) (Fig. 1a–c, Supplementary Fig. 1b, c and Supplementary Data 1). In situ hybridization (ISH) visualized low presence of *hb-egfa* transcripts in uninjured spinal cord, which increased sharply in stump ependymal cells after transection injury, as well as in other cells surrounding the central canal (Fig. 1b and Supplementary Fig. 1b). *hb-egfb* expression was undetectable in uninjured spinal cord but could be detected sparsely in cells throughout the injured cord stumps at 1 and 2 wpi (Fig. 1c and Supplementary Fig. 1c). We assessed transcriptomic datasets for uninjured and 1 wpi cords, finding expression changes in several downstream targets of Hb-egf by pathway analysis of differentially modulated genes. These targets included Akt, the extracellular signal-regulated kinase ERK1/2, and Erbb receptors, previously shown to mediate downstream Hb-egf signaling cascades^35^ (Supplementary Fig. 1a).

To better visualize cell types expressing *hb-egf* after spinal cord injury, we generated reporter lines representing each paralogue. Sham-injured transgenic *hb-egfa:EGFP* fish, generated with ~100 kb BAC sequences spanning the *hb-egfa* locus, displayed little or no detectable enhanced green fluorescent protein (EGFP) expression in cord tissue (Fig. 1d). Upon spinal cord transection, *hb-egfa*-directed EGFP was detected by 1 wpi in cells lining the central canal, mimicking *hb-egfa* ISH patterns (Fig. 1d). At this timepoint, fluorescence largely co-localized with Sox2 and GFAP, markers of ERGs and glia, respectively, and showed indicators of cell cycling at 1 wpi in 5-ethynyl-2′-deoxyuridine (EdU) incorporation assays (Fig. 1e–g and Supplementary Fig. 1d). *hb-egfa*:EGFP partially colocalized with the neuronal marker HuC/D at 1 wpi, with ~10% of Hu/D^+^ cells also expressing EGFP (Supplementary Fig. 1e, f). *hb-egfa:*EGFP fluorescence largely faded by 6 wpi, at which point regeneration typically nears completion (Supplementary Fig. 1g). Interestingly, we found that both *hb-egfa* mRNA and *hb-egfa*:EGFP displayed a polarized distribution, with greater signals in caudal vs. rostral ends (Fig. 1b and Supplementary Fig. 1h, i), a distribution that we did not observe for other genes implicated in spinal cord regeneration like *ctgfa*, *igf*, and *pdgfa* (Supplementary Fig. 1j)^19,36,37^.

We assessed expression of *hb-egfb* by generating an *EGFP* knock-in allele, revealing negligible fluorescence in uninjured *hb-egfb*^*EGFP*^ spinal cords and diffuse EGFP at 1 and 2 wpi (Fig. 1h and Supplementary Fig. 1c). Consistent with ISH, *hb-egfb*^*EGFP*^-directed fluorescence was poorly represented in ependymal cells around the central canal or at sites of tissue bridging (Fig. 1h and Supplementary Fig. 1k). Expression of the potential Hb-egf receptors Erbb4 and Egfr was undetectable in uninjured animals. By 1 wpi, Erbb4 protein was mostly localized in cells at the lesion site. Erbb4 showed little or no expression in ERGs lining the central canal (Fig. 1i). By contrast, *egfra* receptor transcripts were detectable in cells lining the central canal, the spinal cord domain where ERGs reside (Fig. 1j). Thus, *hb-egf* paralogs, receptors, and bioinformatically identified targets are induced after spinal cord injury, with the *hb-egfa* paralog displaying more prominent expression in ERG progenitors.

### Hb-egfa is required for zebrafish spinal cord regeneration

**Fig. 2:**
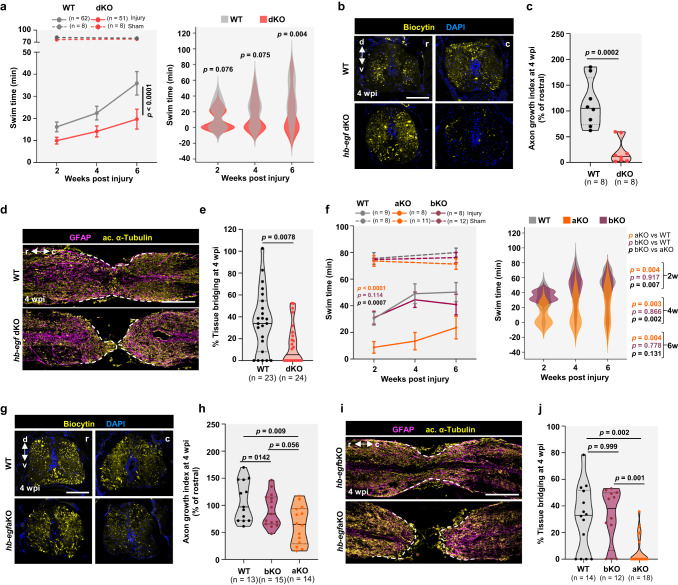
*hb-egfa* paralog is required for zebrafish spinal cord regeneration. **a** Swim capacity of WT (gray) and dKO (red) animals at increasing currents. Data shown as line graphs at increasing timepoints (left) and overlapping violins at each timepoint (right). Two-way repeated-measures ANOVA tests with Holm-Šidák correction. *p* values for sham groups (left graph, dashed lines) are 0.711 and 0.783 at 2 and 6 weeks, respectively. *p* < 0.0001 in left panel represents comparison of performance over all timepoints. **b** Sections of WT and *hb-egf* dKO cords located rostral or caudal to transection site, after anterograde axon tracing at 4 wpi. Quantification in (**c**) *N* = 3. **d** Tissue sections indicating the glial marker GFAP (magenta) and axonal marker acetylated α-Tubulin (yellow) in WT and *hb-egf*dKO spinal cords at 4 wpi. Dashed lines delineate tissue bridging. Quantification in (**e**). *N* = 4. **f** Swim capacity assayed of WT (gray), *hb-egfa* (orange), or *hb-egfb* (dark red) animals. Data shown as line graphs at increasing timepoints (left) and overlapping violins at each timepoint (right). Two-way repeated-measures ANOVA tests with Holm-Šidák correction. *p* values for sham groups (left graph, dashed lines) are 0.730 and 0.236 for aKO vs. WT at 2 and 6 weeks, respectively; 0.862 and 0.681 for bKO vs. WT at 2 and 6 weeks, respectively; 0.830 and 0.562 for aKO vs. bKO at 2 and 6 weeks, respectively. *p* values shown in left graph represent comparison of performance over all timepoints for injured groups. **g** Sections of WT and *hb-egfaKO* spinal cords located rostral or caudal to transection site, after anterograde axon tracing at 4 wpi. Quantification in (**h**). *N* = 2. **i** Tissue sections indicating GFAP (magenta) and acetylated α-Tubulin (yellow) in *hb-egfaKO* or *hb-egfbKO* spinal cords at 4 wpi. Dashed lines delineate tissue bridging. Quantification in (**j**). *N* = 2. Scale bars 200 μm in (**d**, **i**), 100 μm in (**b**, **g**). Error bars in (**a**, **f**) (left graphs) indicate SEM. A two-tailed Mann–Whitney test was used for comparisons in (**c**, **e**, **h**, **j**). r rostral, c caudal, d dorsal, v ventral. *n* = number of animals used for the experiments. Source data provided as Source Data file.

To test requirements for *hb-egf* gene function during spinal cord regeneration, we removed sequences between exons 1 and 4 of *hb-egfa* (referred to as *hb-egfa*KO) and exons 3 and 4 of *hb-egfb* (referred to as *hb-egfb*KO) using CRISPR/Cas9 editing (Supplementary Fig. 2a, b). Animals with mutations in both *hb-egf* paralogues (*hb-egf* dKO) were generated and analyzed first, given the possibility of compensatory effects by gene paralogs that can mask phenotypes in zebrafish^38^. *hb-egf* dKO animals showed no detectable *hb-egf* mRNAs at 1 wpi and are viable to adulthood (Supplementary Fig. 2c, d). These animals showed minimal Sox2^+^ ERG cycling when uninjured like their wild-type clutchmates, assessed by incorporation of 5-ethynyl-2′-deoxyuridine (EdU) (Supplementary Fig. 2e, f), and they displayed grossly normal swim behavior (Fig. 2a and Supplementary Fig. 2g, h).

We subjected mutant animals to spinal cord injury and assessed regeneration at multiple timepoints, at histological and/or functional levels. We first assessed spinal cord function by testing the ability of animals to swim against increasing velocities of water currents. Our experiments revealed measurably reduced swim capacity in *hb-egf* dKO animals in the weeks following spinal cord injury, particularly at the 6 wpi timepoint (Fig. 2a). Notably, just by viewing animals in their standard aquarium setting at 4 wpi, we could discern less frequent or fluid swimming activities in *hb-egf* dKO animals compared with their wild-type clutchmates (Supplementary Movies 1 and 2). Reduced swim capacity was also indicated at 8 wpi (Supplementary Fig. 2i). We then directly assessed axon crossing across the tissue bridge by anterograde labeling, finding that *hb-egf* dKO animals showed ~80% reduction in rostrocaudal axon crossing compared to wild-types at 4 wpi (Fig. 2b, c). We finally measured tissue bridges, comprising both glia and neurons, by staining longitudinal sections of *hb-egf* dKO and wild-type spinal cords at 4 wpi for glial and axonal markers. The average diameter of bridges was reduced by ~59% in *hb-egf* dKO animals compared to wild-type clutchmates (Fig. 2d, e).

**Fig. 3:**
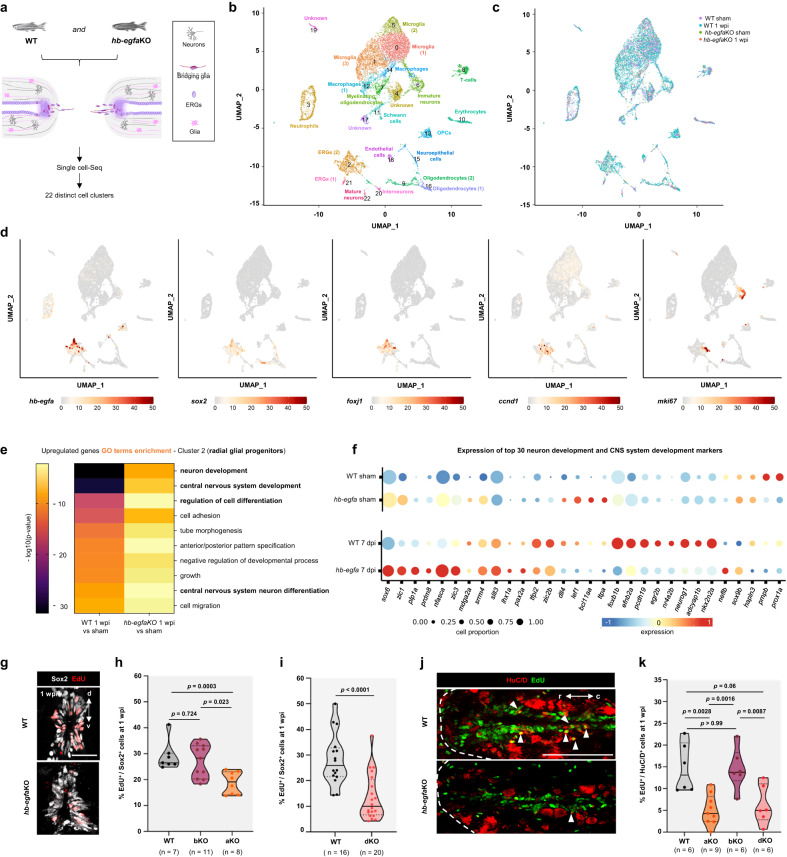
scRNA-seq identifies a role for Hb-egfa in stump neurogenesis during spinal cord repair. **a** Diagram with scRNA-Seq strategy. **b** UMAP showing clustering of scRNA-Seq data at 1 week post injury (wpi). **c** Cells from sham-injured and 1 wpi wild-type (WT) and *hb-egfa*KO spinal cords contribute similarly to all identified cell clusters. **d** Expression of *hb-egfa* and ERG marker genes in ERGs at 1 wpi. **e** Heatmap showing enriched GO terms at 1 wpi in radial glial progenitors of WT and *hb-egfaKO* spinal cords. **f** Expression of genes involved in neuronal differentiation and central nervous system development identified by GO analyses in WT and *hb-egfaKO* spinal cords. Dot size represents the percentage of positive cells for each gene, and dot color represents the expression level. Top 30 genes ordered by *p* value are shown. **g** Transverse sections of WT and *hb-egfaKO* spinal cords at 1 wpi, stained for ependymal cells (Sox2^+^, white) and EdU (red) incorporation. **h** Quantification of Sox2^+^ ependymal cell cycling at 1 wpi in WT, *hb-egfaKO, or hb-egfbKO* fish. *N* = 2. **i** Quantification of cycling ependymal cells in WT and *hb-egf* dKO spinal cords at 1 wpi. *N* = 3. **j** Longitudinal sections of WT and *hb-egfaKO* spinal cords at 1 wpi, stained for the neuronal marker HuC/D (red) and the cycling marker EdU (green). Arrowheads indicate EdU-labeled cells, and dashed line delineates the spinal cord stumps. **k** Quantification of neurons with EdU labeling in WT, *hb-egfaKO*, *hb-egfbKO* and *hb-egf* dKO cords at 1 wpi. *N* = 3. Scale bars 50 μm in (**g**), 200 μm in (**j**). A two-tailed Mann–Whitney test was used for comparisons in (**h**, **i**, **k**). r rostral, c caudal, d dorsal, v ventral. *n* = number of animals used for the experiments. Source data are provided as a Source Data file.

To assess which paralog(s) has an important function in spinal cord regeneration, we performed the same assays in *hb-egfa* and *hb-egfb* single mutants. Swim performance after spinal cord injury was reduced in *hb-egfa*KO animals compared to controls, with swim behavior in aquaria at 4 wpi noticeably different from wild-types, as observed with *hb-egf* dKO animals. Swim capacity in *hb-egfb*KO mutants was comparable to wild-types (Fig. 2f and Supplementary Movies 3 and 4). Correspondingly, *hb-egfaKO* animals showed a ~41% decrease in rostrocaudal axon crossing compared to wild- types at 4 wpi, whereas *hb-egfbKO* animals displayed no measureable defects (Fig. 3g, h). Furthermore, *hb-egfb*KO animals displayed similar tissue bridging compared to wild-types after spinal cord injury. By contrast, we observed a ~80% reduction in tissue bridging in *hb-egfa*KO zebrafish (Fig. 2i, j). Thus, *hb-egf* gene knockout delays and/or disrupts spinal cord regeneration, with our expression data and mutant analysis indicating Hb-egfa is the more crucial gene product of the two paralogs during spinal cord regeneration.

### Impaired neurogenesis underlies defects in *hb-egfa* mutants

To initially chart the phenotype of *hb-egfa* mutants, we performed single-cell RNA sequencing (scRNAseq) profiling of wild-type and *hb-egfa*KO spinal cords that were sham-injured or at 1 wpi (Fig. 3a and Supplementary Fig. 3a–c). Unsupervised clustering of 10,652 cells revealed 22 cell clusters, including major spinal cord cell types, endothelial cells, and immune cells, identifiable by specific marker genes (Fig. 3b). Cell cluster composition was similar in the 4 groups (Fig. 3c), and no substantial differences were observed in the proportion of each cell type identified between wild-type and *hb-egfa*KO samples at this early stage (Supplementary Fig. 3d). *hb-egfa* was most enriched in ERG progenitor clusters, identified by expression of *sox2*, *foxj1a, ccnd1* and *mki67* (Fig. 3d and Supplementary Fig. 3f), and it was also expressed at low levels in other cell types including oligodendrocyte progenitors, neutrophils, neuroepithelial cells and mature neurons (Supplementary Fig. 3e).

To assess intercellular communication networks involving Hb-egf, we used CellChat to detect potential ligand-receptor pairing among the 22 cell clusters (Supplementary Fig. 3g). This analysis identified 81 signaling pathways showing activity during spinal cord regeneration, including those previously implicated like Tnf, Pdgf, Sema, Tenascin and Igf signaling^23,36,37,39,40^ (Supplementary Fig. 3h). Oligodendrocyte precursors displayed the most predicted signaling interactions, followed by mature oligodendrocytes, radial glial progenitors, interneurons and mature neurons (Supplementary Fig. 3h). Notably, EGF signaling was among the most represented pathways in wild-type samples at 1 wpi, most often as interactions between Hb-egfa ligand (produced predominantly but not exclusively by ERGs) and its Egfra receptor signaling in a variety of cell types (Supplementary Fig. 3i). Cell types interpreted as major influencers controlling Hb-egfa communication were endothelial cells, neutrophils, oligodendrocyte precursors, and radial glial progenitors, the last two of which were also interpreted as mediators (Supplementary Fig. 3j, k).

To assess effects of Hb-egfa deficiency on ERGs, we first performed Gene Ontology comparisons of expression signatures of wild-type and *hb-egfa*KO ERGs. *hb-egfa* mutant cells displayed limited induction of genes associated with neuron development and differentiation at 1 wpi, compared to the responses of wild-type ERGs (Fig. 3e, f). Analysis of canonical pathways revealed highest enrichment of components involved in Synaptogenesis and Axon Growth in 1 wpi wild-type ERGs, whereas pathways related to Fibrotic events were most enriched in *hb-egfa*KO ERGs (Supplementary Fig. 3l, m). Injury-associated increases in levels of neuronal markers such as *neuroD4*, *sox11b* and *adcyap*, as assessed by qPCR, were greater in wild-type spinal cords vs. *hb-egfa*KO cords at 1 wpi and 2 wpi (Supplementary Fig. 3n).

The dominant expression domains of *hb-egfa* by all expression assays were ERGs and their 2 major derivatives, glia and HuC/D^+^ neurons, in that order. To visualize ERG behavior in situ, we assessed cycling of Sox2^+^ cells at 1 wpi by EdU incorporation or PCNA immunostains. These experiments indicated a ~35% reduction in ERG cycling in *hb-egfa*KO animals as compared to wild-type clutchmates (Fig. 3g, h and Supplementary Fig. 4a, b). *hb-egf* dKO animals displayed a similar, ~52% reduction in Sox2^+^ ERG cycling compared to wild-types, whereas *hb-egfb*KO values were comparable to controls (Fig. 3g–i and Supplementary Fig. 4a, b). To test effects of *hb-egf* mutations on gliogenesis, which could occur by ERG differentiation or by direct glial cell division, we measured EdU incorporation in GFAP^+^ cells. We observed a ~52% reduction in cycling in injured *hb-egf* dKO cords compared to controls at 1 wpi (Supplementary Fig. 4c, d). Most cycling glial cells at this time were located dorsally around the central canal (Supplementary Fig. 4c). *hb-egfa*-directed fluorescence partly co-localized with a transgenic marker for the bridging glial marker *ctgfa* at 2 wpi (Supplementary Fig. 4e), as would be predicted by recent single-cell RNA-seq datasets^41^. Yet, there was no detectable difference in *ctgfa* mRNA expression patterns in *hb-egf*dKO cords, or in *hb-egfa* mRNA expression patterns in *ctgfa* mutant cords, that might have suggested a regulatory relationship (Supplementary Fig. 4f, g).

To visually assess neurogenesis to stages beyond ERG cell cycle entry, we performed spinal cord transection in mutant animals and measured EdU incorporation into cells expressing the neuronal marker HuC/D^+^ in regions near the lesion at 1 wpi. This assay indicated ~57% and ~65% reductions in HuC/D^+^ neuron labeling in *hb-egf* dKO and *hb-egfa*KO bridging sites, with no detectable difference between *hb-egfb*KO and control values (Fig. 3j, k). EdU incorporation indices into rostral and caudal neurons were similar, with decreases from wild-types in each domain in *hb-egfa*KO and *hb-egf* dKO cords (Supplementary Fig. 4k). EdU incorporation into HuC/D^+^ cells was similar between wild-type, *hb-egfaKO*, *hb-egfbKO*, and *hb-egf* dKO at a later stage of 2 wpi (Supplementary Fig. 4l). Axon regeneration was grossly normal in *hb-egf* dKO larvae after severing a small bundle of spinal cord axons, suggesting that Hb-egfa does not have a direct role to promote axon crossing during spinal cord regeneration (Supplementary Fig. 4m, n). For insights into mechanisms by which Hb-egfa supports neurogenesis at early stages after injury, we used Panther^42^ to perform statistical overrepresentation tests using the list of genes differentially modulated at 7 dpi in *hb-egf* dKO ERGs compared to *hb-egf* WT ERGs. Interestingly, genes involved in Notch and Wnt signaling pathways were over-represented in the gene list, with reductions in expression levels of genes implicated in these pathways in *hb-egfaKO* ERGs vs. controls. These same signaling pathways and genes were implicated as downstream effectors of Hb-egfa in a previous study of Muller glia during retinal regeneration^32^ (Supplementary Fig. 4o–q).

Taken together, our transcriptomics, EdU labeling and bioinformatic assays indicate that Hb-egfa has an early and essential role in producing key ERG derivatives including neurons at early stages upon spinal cord injury, enabling long-term recovery of motor function.

### Local HB-EGF application improves spinal cord regeneration

**Fig. 4:**
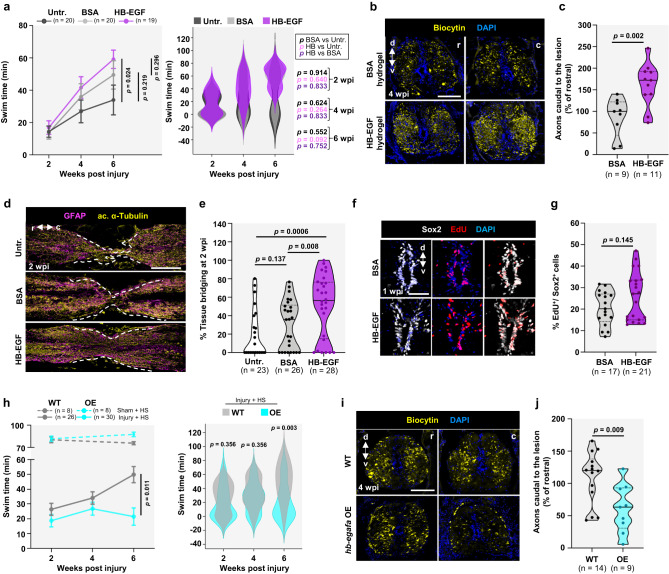
Effects of Hb-egf supplementation on spinal cord regeneration. **a** Swim tests in fish treated with HR-HB-EGF hydrogel (purple), BSA hydrogel (gray), or untreated (charcoal gray) at the site of a spinal cord crush injury. Two-way repeated-measures ANOVA tests with Holm-Šidák correction were used for comparisons. **b**, **c** Cross sections of BSA- and HR-HB-EGF-hydrogel-treated spinal cords located rostral and caudal to the transection site, after anterograde axon tracing at 4 wpi. Quantification shown in (**e**). *N* = 4. A two-tailed unpaired *t*-test with Welch’s correction was used for comparison. **d** Longitudinal sections indicating GFAP (magenta) and acetylated α-Tubulin (red) immunofluorescence at 2 wpi in fish treated with BSA- or HR-HB-EGF-loaded hydrogel. Dashed lines delineate sites of tissue bridging. *N* = 4. **e** Quantification of tissue bridging. Mann–Whitney tests were used for comparisons. *N* = 3. **f**, **g** Ependymal cell cycling assessed by EdU (red) incorporation in spinal cords of fish treated with vehicle (BSA)- or HR-HB-EGF-loaded hydrogel, shown as transverse sections at 1 wpi. Quantification shown in (**c**). *N* = 3. A two-tailed Mann–Whitney test was used for comparisons. **h** Swim capacity assayed in WT (gray, full line) or *hb-egfb*OE (cyan, full line) animals at 2, 4 and 6 wpi, and in uninjured animals (dashed lines). Whole-animal *hb-egfa* overexpression impairs recovery. Two-way repeated-measures ANOVA tests with Holm-Šidák correction were used for comparisons. *p* values for sham groups in left graph, dashed lines, are *p* = 0.764 at 2 weeks and *p* = 0.028 at 6 weeks. *p* = 0.011 shown in left panel represents comparison of performance over all timepoints. **i**, **j** Cross sections of wild-type (WT) and *hb-egfa*OE spinal cords located rostral or caudal to the transection site, after anterograde axon tracing at 4 wpi. Quantification shown in (**h**). *N* = 4. A two-tailed unpaired *t*-test with Welch’s correction was used for comparison. Scale bars 50 μm in (**f**), 200 μm in (**d**), 100 μm in (**b**, **i**). Error bars in (**a**, **h**) indicate SEM. r rostral, c caudal, d dorsal, v ventral. *n* = number of animals used for the experiments. Source data are provided as a Source Data file.

To assess effects of a targeted increase in Hb-egf gene products, we implemented a biomaterial-assisted strategy using a hyaluronic acid (HA)-based dynamic hydrogel^43^. Injection of a FITC-loaded version adjacent to the region of spinal cord transection resulted in gradual diffusion from the injection site, and fading by 21 days post injection (Supplementary Fig. 5a). To supplement Hb-egf at injury sites, we injured animals and immediately applied HA-hydrogels containing either BSA (control) or embedded human recombinant (HR) HB-EGF protein. Both BSA and HR-HB-EGF hydrogel groups showed an improvement in swim capacity compared to untreated fish, consistent with a beneficial role of hydrogel scaffolds at the site of spinal cord injury as shown in mammals^44,45^, with swim capacity trending higher in the HB-EGF group (Fig. 4a). Localized delivery of HR-HB-EGF resulted in a ~46% increase in the average axon crossing index at 4 wpi (Fig. 4b, c) and a ~40% increase in the average diameter of tissue bridges at 2 wpi relative to controls (Fig. 4d, e). Values of ependymal cell cycling and dorsal glial cycling trended higher in HR-HB-EGF-treated animals at 1 wpi (Fig. 4f, g and Supplementary Fig. 5b, c). We also found that administration of HR-HB-EGF was sufficient to improve cycling in *hb-egf* dKO Sox2^+^ ERGs at 1 wpi, and tissue bridging and axon crossing at later stages, indicating specificity of the phenotypes as well as the treatment (Supplementary Fig. 5d–g).

We also generated transgenic fish expressing a soluble form of Hb-egfa under the control of a heat-shock-inducible promoter (*hsp70*:*hb-egfa-2A-TBFP*, referred to as *hb-egfaOE*). Daily heat shocks caused whole-animal overexpression visualized by TBFP fluorescence (Supplementary Fig. 5h, i). We injured animals subjected to this heat-shock regimen and examined features of spinal cord regeneration. We found that daily, animal-wide induction of *hb-egfa* grossly impaired recovery of swim capacity (Fig. 4h and Supplementary Movies 5 and 6), and caused a ~44% decrease in axon crossing indices at 4 wpi (Fig. 4i, j). ERG cycling and tissue bridging were not grossly affected (Supplementary Fig. 5j, k). Thus, higher levels of Hb-egf throughout the entire cord and surrounding tissues do not improve regeneration, but instead can adversely affect functional regeneration. Together, our gain-of-function experiments suggest that Hb-egf has instructive rather than permissive effects on spinal cord regeneration; i.e. it can modulate regeneration when expressed ectopically, with local augmentation improving indicators of regeneration.

### *hb-egfa*-linked enhancer directs expression to cord injuries

**Fig. 5:**
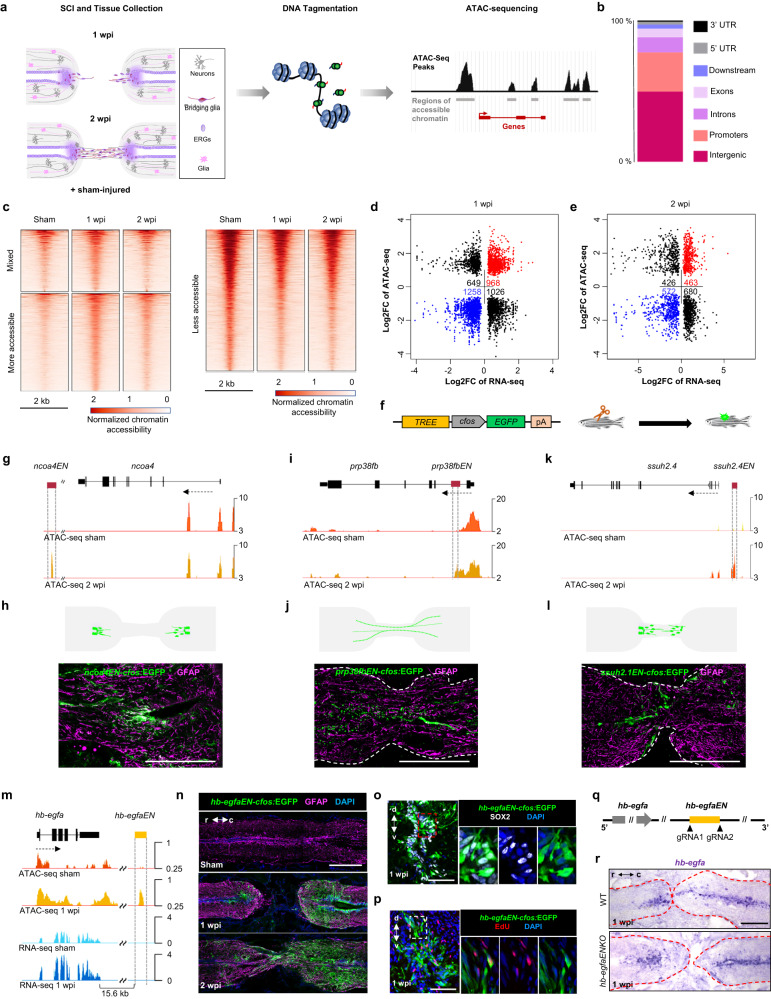
Chromatin profiling reveals an enhancer linked to *hb-egfa* that directs regeneration-associated gene expression. **a** Diagram with strategy used to identify spinal cord TREEs. **b** Bar plot showing the proportions of dynamic peaks during regeneration located within promoters, exons, introns, and intergenic regions. **c** Heat map of ATAC-seq signals in sham-injured, 1 and 2 weeks post injury (wpi) spinal cord tissue. Mixed peaks show different trends in 1 and 2 wpi samples, compared with the control. Cutoff is *p* value < 0.05 and fold change >1.2. Dot plot of differential ATAC-seq chromatin regions linked to nearby differential transcripts at 1 (**d**) and 2 wpi (**e**) vs. sham-injured spinal cords. Each point indicates a separate ATAC-seq peak. **f** Transgene used to assess in vivo TREE function. **g**, **i**, **k** Browser tracks indicating chromatin accessibility (dark and light orange) at the *ncoa, prp38fb and ssuh2.4* locus. The candidate *ncoa*-linked enhancer *ncoaEN, prp38fb*-linked enhancer *prp38fbEN* and *ssuh2.4*-linked enhancer *ssuh2.4EN* are indicated with dashed lines. **h**, **j**, **l** Cartoon and immunofluorescence images showing injury-induced expression patterns directed by *ncoaEN, prp38fb EN and ssuh2.4EN* at 2 weeks post injury. **m** Browser tracks indicating chromatin accessibility (dark and light orange) and transcript levels (light and dark blue) at the *hb-egfa* locus, indicating the candidate *hb-egfa*-linked enhancer *hb-egfaEN* with dashed lines. *hb-egfaEN* is located 15.6 kb downstream of *hb-egfa* and increases accessibility during regeneration. **n** Longitudinal sections of *hb-egfaEN-cfos:EGFP* spinal cords showing induced EGFP at 1 and 2 wpi. Transverse section images indicating immunofluorescence for Sox2 (**o**) and EdU incorporation assay for cell cycling (**p**) in cells displaying *hb-egfEN*-directed EGFP expression at 1 wpi. **q** Strategy used to generate *hb-egfaEN* mutants. **r** In situ hybridization indicating grossly normal expression of *hb-egfa* mRNA in spinal cords of wild-type (WT) and *hb-egfaEN* mutant fish at 1 wpi. *N* = 2 in (**h**, **j**, **l**); *N* = 3 in (**n**–**p**, **r**). Scale bars 200 μm in (**h**, **j**, **l**, **n**, **r**), and 50 μm in (**o**, **p**). r rostral, c caudal, d dorsal, v ventral.

Regeneration programs are orchestrated at least in part by gene regulatory elements named “tissue regeneration enhancer elements” (TREEs)^46,47^. These enhancers possess sequences that direct gene expression preferentially or specifically upon injury to the damaged area, maintain expression during regeneration, and allow expression to temper as regeneration concludes. We hypothesized that one or more DNA regulatory elements responsible for *hb-egfa* expression is located in sequences represented in the *hb-egfa* BAC used to generate transgenic reporter animals. To identify TREEs relevant to spinal cord regeneration, we employed Assay for Transposase-Accessible Chromatin using sequencing (ATAC-seq), a genome-wide assay of chromatin accessibility^48^ (Fig. 5a). From this assay, we identified 3901 and 2141 sequences changing accessibility at 1 and 2 wpi, respectively, most of which were in intergenic regions (49.5%), in promoters (27.7%) or within introns (10.9%). The remaining regions were located in UTRs, exons or immediate downstream regions of genes (Fig. 5b, c). We then bioinformatically assigned regions identified by ATAC-seq to their closest transcriptional start site and integrated them with RNA-seq transcriptomic data (1 wpi newly generated, 2 wpi from^19^; Fig. 5d, e and Supplementary Data 2 and 4). Gene ontology analyses of chromatin peaks associated with nearby genes revealed enrichment of pathways involved in developmental growth, with major functions in central nervous system development, axonogenesis and axon guidance, neurogenesis, and angiogenesis at 1 and 2 wpi, supporting the establishment of regeneration programs at early stages after injury (Supplementary Fig. 6a, c and Supplementary Data 3 and 5). We found 968 and 463 regions increasing accessibility after spinal cord injury and associated with upregulated genes during spinal cord regeneration at 1 and 2 wpi, respectively (Fig. 5d, e, red quarter and Supplementary Fig. 6b, d), potentially acting as TREEs. Several regulatory sequences were tested for enhancer activity in stable transgenic lines when encompassed upstream of the minimal promoter *c-fos* and an *EGFP* reporter gene (Fig. 5f). For example, separate sequences linked to *ncoa4*, *prp38fb* and *ssuh2.1* each directed injury-responsive expression in spinal cords, showing differential tissue representation (Fig. 5g–l).

Interestingly, one of these dynamically accessible sequences was a 310 bp region located 15.6 kb downstream of the *hb-egfa* start site with increased accessibility at 1 wpi as compared to sham-injured spinal cords (referred to as *hb-egfaEN*, Fig. 5m). To test whether *hb-egfaEN* can direct injury-induced gene expression after spinal cord injury, we established transgenic lines as above (Fig. 5f). Whereas we did not detect spinal cord fluorescence in uninjured *hb-egfaEN-cfos:EGFP* animals or in animals carrying a *cfos:EGFP* control construct (Fig. 5n and Supplementary Fig. 6e), *hb-egfaEN* directed EGFP expression at spinal cord injury sites (3 stable lines assessed; Fig. 5n). *hb-egfaEN-cfos:*EGFP had very similar spatiotemporal dynamics after spinal cord injury as those in *hb-egfa:EGFP* BAC transgenics, with greater fluorescence in caudal vs. rostral stumps at 1 and 2 wpi, respectively (Fig. 5n and Supplementary Fig. 6f). Cells lining the central canal, including those positive for Sox2 and indicators of cell cycling, displayed *hb-egfaEN-*directed expression at 1 wpi (Fig. 5o, p). Fluorescence diminished by 4 wpi, with only limited expression evident in some animals at 6 wpi (Supplementary Fig. 6g). To identify potential upstream regulators, we performed bias-free transcription factor footprint enrichment-test (BiFET) sequencing analyses, which revealed over-representation of predicted binding motifs for Rreb, Znf317, Tcf3 and Hoxb5 (Supplementary Fig. 6i).

We examined the requirement of *hb-egfaEN* for *hb-egfa* expression by using CRISPR/Cas9 to excise a ∼1.2 kb DNA region encompassing *hb-egfaEN* (Fig. 5q), which did not detectably alter expression of *hb-egfa* after transection injury (Fig. 5r). Enhancer redundancy is common (reviewed in ref. ^49^), and we suspect that other regulatory sequences compensate to maintain *hb-egfa* expression in *hb-egfaEN* deletion mutants. To begin to address this, we examined *hb-egfa* upstream and downstream sequences for predicted TF binding motifs. Sox2 binding sites were enriched in these regions (Supplementary Fig. 6j), and, given that *hb-egfa* expression is induced in Sox2^+^ ERGs, we hypothesized that Sox2 regulates *hb-egfa* expression. Consistent with this idea, we found that induced overexpression of Sox2^50^ is sufficient to increase *hb-egfa* transcript levels and *hb-egfa* BAC-directed EGFP fluorescence in larvae (Supplementary Fig. 6k, l). In contrast, Sox2 overexpression did not change EGFP expression in *hb-egfaEN-cfos:EGFP* zebrafish (Supplementary Fig. 6m). Together, our results provide evidence that *hb-egfaEN* is a TREE that directs one or more components of *hb-egfa* expression during spinal cord regeneration, and that *hb-egfa* regulation is likely complex, with key Sox2-responsive and -nonresponsive elements.

### *hb-egfaEN* directs expression in neonatal mouse spinal cords

**Fig. 6:**
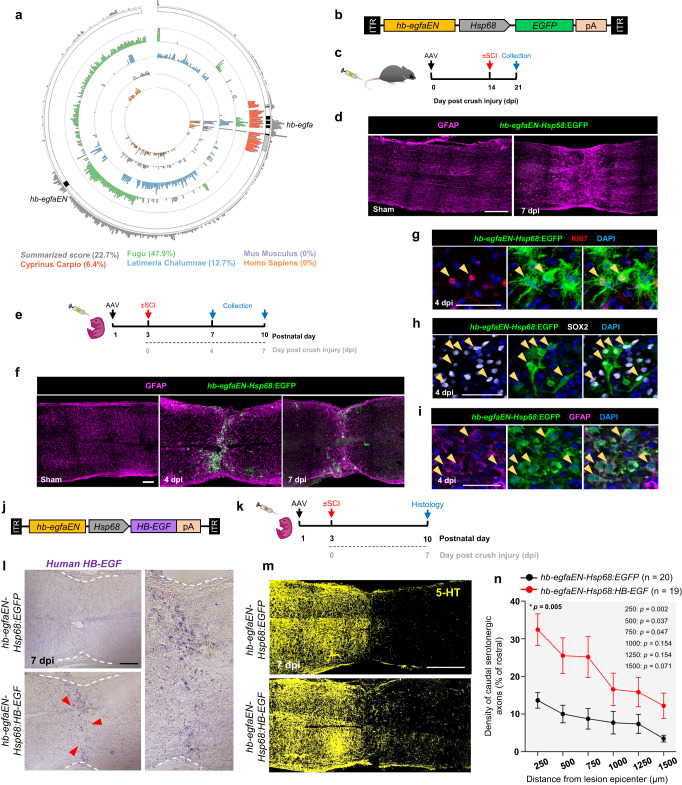
*hb-egfaEN* directs injury-associated gene expression in neonatal mice and improves axon density through HB-EGF delivery. **a** Circle plot showing conservation of zebrafish *hb-egfaEN* in different species. Percentage values indicate conservation scores. Summarized score is the average of the similarity scores of species shown. **b** Viral construct to evaluate the ability of zebrafish *hb-egfaEN* to direct expression in mouse spinal cord upon crush injury. **c** Experimental design to test *hb-egfaEN* activity in adult mouse spinal cord after systemic delivery of an AAV vector. **d** Longitudinal sections of spinal cord in sham-injured adult mice and at 1 wpi. **e** Experimental design to test *hb-egfaEN* activity in neonatal mouse spinal cord after systemic viral delivery. **f** Longitudinal sections of neonatal mouse spinal cords, either sham-injured or at 4 or 7 dpi (crush). *N* = 3. Expression of the marker of cycling Ki67 (**g**), the transcription factor Sox2 (**h**), and the glial marker GFAP (**i**) in cells displaying *hb-egfaEN*-directed EGFP expression at 4 dpi (crush) in neonatal spinal cord. *N* = 2. **j** Viral construct and (**k**) experimental design to concentrate expression of human HB-EGF at the lesion site of neonatal spinal cords. **l** In situ hybridization indicating expression of human *HB-EGF* mRNA at 7 dpi (crush) in mice transduced with AAV carrying *hbegfaEN-Hsp68:EGFP* (top panel) or *hbegfaEN-Hsp68:HB-EGF* (bottom panel) constructs. Magnified panel on the right shows site of injury in *hbegfaEN-Hsp68:HB-EGF* mice. Red arrows indicate *HB-EGF* mRNA signal. *N* = 2. **m** Longitudinal sections of crush-injured spinal cords of neonatal mice at 7 dpi, stained for the marker of serotonergic axons, 5-HT. Sections from 20 *hb-egfaEN-Hsp68:EGFP* and 19 *hbegfaEN-Hsp68:HB-EGF* treated mice were analyzed from two independent experiments. Quantification of the density of caudal serotonergic axons at 7 dpi shown in (**n**). Two-way repeated-measures ANOVA tests with Holm-Šidák correction were used for comparisons. *p* = 0.005 shown in upper left represents comparisons over all distances. Scale bars 500 μm in (**d**, **m**), 200 μm in (**l**), 100 μm in (**f**), 50 μm in (**g**), 40 μm in (**h**, **i**). Error bars in (**n**) indicate SEM. *n* = number of animals used. Source data are provided as a Source Data file.

Recent reports have hypothesized that the acquisition, retention, or loss of TREEs might contribute to differences in regenerative capacity among species^47,51^. Moreover, we recently reported that zebrafish TREEs can activate injury induced expression when introduced in adult mammals^52^. We examined several vertebrate genomes for *hb-egfaEN* sequences, finding significant primary sequence identity only in *Fugu* (Fig. 6a). To test for functional conservation in mammals, we generated a recombinant adeno-associated viral vector (AAV) containing zebrafish *hb-egfaEN* upstream of a murine permissive *Hsp68* promoter and an *EGFP* reporter gene (Fig. 6b). To maximize payload expression in spinal cord tissue, we used an AAV9-based capsid variant (AAV.cc47^53^) that we found to effectively transduce different spinal cord cell types when delivered systemically by tail vein injection to adult mice (Supplementary Fig. 7a–c). To assess if zebrafish *hb-egfaEN* can direct injury-induced expression in mice similarly as in zebrafish, we first injected 3-month-old adult mice with AAV-*hb-egfaEN-Hsp68:EGFP* construct, performed thoracic crush injuries (T9-T10 level) 2 weeks after injection, and assessed EGFP fluorescence at 1 wpi (Fig. 6c). Thoracic crush injuries are commonly used in mammals to model the human condition, and we also observed that *hb-egfa* and *hb-egfaEN*-directed EGFP are induced in zebrafish after crush injuries (Supplementary Fig. 6h). We observed little or no EGFP in spinal cords of adult mice treated with AAV-*hb-egfaEN-Hsp68:EGFP* or control AAV*-Hsp68:EGFP* in sham or crush injuries (Fig. 6d and Supplementary Fig. 7d–f), nor was EGFP observed after a severe transection injury (Supplementary Fig. 7g–i).

Likely explanations for differential cross-species reading of *hb-egfaEN* include: (1) changes in *hb-egf* linked non-coding sequences during evolution rendered zebrafish sequences unrecognizable by mammalian transcriptional machinery; and/or (2) relevant transcriptional activators are not present or active in injured spinal cord tissues of adult mice. To attempt to resolve this, we introduced these same vectors at the more regeneration-competent neonatal stages of mice. First, we performed spinal cord crush injury in P3 pups and assessed the extent of axon growth across lesions at 1 and 14 dpi. We observed serotonergic axons distal to the injury and modest GFAP accumulation at the lesion site by 14 dpi, indicating similar regenerative capacity as reported^3^ (Supplementary Fig. 8). After observing that our AAV9-based capsid variant effectively transduced spinal cord cell types in neonates as in adults (Supplementary Fig. 7k–m), we systemically administered AAV*-hb-egfaEN-Hsp68:EGFP* or control AAV-*Hsp68-EGFP* vectors at P1, subjected animals to crush injuries at P3 (T9-T10 level), and analyzed tissue at two timepoints after injury (Fig. 6e). AAV-*Hsp68:EGFP* directed occasional but minimal EGFP expression in sham or crush injuries, similar to experiments in adults (Supplementary Fig. 7n–p). Notably, neonatal mice transduced with AAV-*hb-egfaEN-Hsp68:EGFP* displayed a prominent and restricted EGFP domain in 4 and 7 dpi injury sites (Fig. 6f). *hb-egfaEN*:EGFP^+^ cells lined the lesion site and expressed markers characteristic of zebrafish ERGs like Sox2 and GFAP at 1 wpi, with many of these cells also positive for the cycling marker Ki67 (Fig. 6g–i). Most EGFP^+^ cells did not express markers of neurons (HuC/D), macrophages (F4/80), or microglia (CD68) at 4 dpi, nor did they express Fibronectin, a component of bridges in neonatal mice (Supplementary Fig. 9). We conclude that the injury-targeting instructions of the zebrafish TREE *hb-egfaEN* can be interpreted by murine transcription complexes, but that key components of this recognition are present only at early life stages and lose efficacy with age.

### HB-EGF gene therapy improves axon density in neonatal mice

To test whether Hb-egf has instructive effects on regeneration after spinal cord injury in mice, we established a strategy harnessing *hb-egfaEN* to augment levels of human HB-EGF in the lesion sites of neonatal mice. Analysis of published transcriptomes^3^ indicated that *Hb-egf* RNA is expressed in murine astrocytes, where its levels increase upon injury (Supplementary Fig. 10a, b). We generated recombinant AAV vectors packaging the zebrafish *hb-egfaEN* upstream of an *Hsp68* minimal promoter and a gene cassette encoding a constitutively secreted form of human HB-EGF (AAV9-*hbegfaEN-Hsp68:HB-EGF*) (Fig. 6j). Following systemic administration of this vector or an AAV-*hbegfaEN-Hsp68:EGFP* control vector at P1 and thoracic crush injuries at P3 as above, we analyzed spinal cord tissue histologically at P10 (Fig. 6j–l). As expected, zebrafish *hb-egfEN* was able to concentrate human *HB-EGF* mRNA at 7 dpi lesion sites in mice pre-treated systemically with experimental virus (Fig. 6l). To determine if the targeted HB-EGF payload altered responses to spinal cord injury, we quantified the density of serotonergic axons caudal to the crush site at 7 dpi. We observed increases in the numbers of serotonergic axons downstream of lesions in AAV-*hbegfaEN-Hsp68:HB-EGF*-transduced animals, measured at various distances from the trauma (Fig. 6m, n). We monitored these mice for functional recovery up to 4 wpi, finding a modest degree of recovery in hind-limb stepping and coordination, consistent with previous studies^3^, without gross differences between EGFP and HB-EGF groups (Supplementary Movies 7 and 8). Thus, by use of an enhancer sequence that directs injury-responsive expression during innate regeneration in zebrafish, we find that a spatiotemporally controlled dose of HB-EGF can improve an indication of spinal cord repair in mice.

## Discussion

By generating stable genetic reagents for *hb-egf* genes and assessing a full panel of spinal cord regeneration assays, we propose a model in which Hb-egfa produced in ERGs at spinal cord injury sites promotes ERG cycling and neuron production, a neurogenic role that antecedes tissue bridging, axon crossing and functional recovery (Supplementary Fig. 5l). Few factors have yet been implicated in the process of neurogenesis during adult spinal cord regeneration, among which are Hedgehog^54^, Notch^55^, FGF^56^, dopamine^57^, serotonin^58^ and myostatin^59^. Inducible fate-mapping studies will be necessary to further define how the newly generated neurons integrate into and help construct the bridge, as will methods to visualize new synaptic connections and circuit formation. It is possible that Hb-egf-associated neurogenesis at injury stumps might also contribute more directly to reestablishing functional synaptic connections. Other factors such as Neurotrophin-3 have been shown to induce generation of spinal cord neurons at the lesion site after transection injury in rats. Over the long term, these neurons have been shown to interconnect severed ascending and descending axons, resulting in sensory and motor behavioral recovery^60^.

We identify an enhancer (*hb-egfaEN)* that directs expression of a reporter gene in response to spinal cord injury, with a pattern mimicking that of *hb-egfa* itself that includes a caudal expression bias. Deletion of this enhancer sequence does not alter the *hb-egfa* expression pattern, a feature that we have reported for other TREEs^61,62^ and in this case might reflect Sox2-dependent and -independent regulation. We find that zebrafish *hb-egfaEN* sequences can functionally interact with mammalian transcriptional machinery after spinal cord injury, a finding that is not entirely surprising based on our recent examination of zebrafish TREEs in other injury contexts in mammals^52^. Yet, intriguingly, our data indicate that cross-species recognition of *hb-egfaEN* is specific to the context of the neonatal stage. Whereas spinal cord injury in adult mice triggers the formation of a scar consisting of cells like reactive astrocytes, fibroblasts, microglia and macrophages, neonatal mice have a more permissive, pro-regenerative environment with limited scarring after spinal cord injury^63^. One aspect of the neonatal response is the construction of fibronectin bridges by microglia, which revert to their homeostatic state after exerting pro-regenerative functions^3^. The contextual, age-dependent recognition *of hb-egfaEN* we observe here hints at a potentially broad concept in which regenerative capacity can be defined in part by the ability to open, read, and follow the instructions of key regeneration-activated regulatory sequences. This ability might be founded on context-dependent chromatin environments, which have been affiliated with regeneration and early development in previous studies^64,65^, and/or with selective presence of central transcription factors and their ability to bind key TREEs. Further comparisons of chromatin structure and transcription complexes in mammalian spinal cord tissue at different life stages, including their ability to recognize additional TREEs, are likely to provide helpful perspective about the developmental loss of pro-regenerative capacity with aging.

Finally, we report here that human HB-EGF delivery via a gene therapy vector improves axon density downstream of injury sites after spinal cord crush injury in neonatal mice. Whether this occurs because of neurogenesis-related effects, as observed in zebrafish, or other potential mechanisms such as neuroprotection, remain to be investigated. In our experiments, although the experimental viral vector was delivered systemically, inclusion of *hb-egfaEN* in viral sequences focused *HB-EGF* expression to the injury site with temporal control. This element of control is attractive for potential therapies, given that the extent of neurogenesis and instructions for axons are likely to require some level of spatiotemporal precision. HB-EGF itself has been proposed to be tumorigenic, thus requiring spatiotemporally targeted delivery if used therapeutically^66^. There are many components of this delivery system that can be optimized with the intent to alter cell-type specificity, temporal dynamics, dose, and off-target effects. With such optimization, TREE-based delivery systems for factors like HB-EGF have potential applications in precision interventions to enhance regenerative responses after injury to the spinal cord.

## Methods

### Zebrafish

Wild-type, mutant, or transgenic male and female zebrafish of the Ekkwill (EK) strain were used for all experiments. Adult animals were between 3 and 12 months of age, with the majority between 6 and 12 months, and measured ~2.5 cm in length. Animals were maintained at a density of ~15–18 fish per 3 liters. Larvae used for experiments were between 3 and 6 days post fertilization. The following previously published lines were used for this study: *Tg(gfap:EGFP*^*mi2002*^*)*^67^, *Tg(−5.5Kb-ctgfa:EGFP*^*pd96*^*)*^19^*, ctgfa*^*bns5*19^, and *Tg*(*hsp70:sox2*^*x21*^*)*^50^. Transection and crush injuries were performed midway between the brainstem and the base of the caudal fin. Clutchmates were used as controls for all experiments. Experiments with zebrafish were approved by the Institutional Animal Care and Use Committee (IACUC) at Duke University. Animals in control and experimental groups received the same care, food and follow up for the duration of the experiments.

#### Generation of *hb-egfa:EGFP* zebrafish

To generate the *hb-egfa:EGFP* BAC construct, the first exon of the *hb-egfa* gene in the BAC clone CH73-26l13 (containing ~66 kb upstream and 31 kb downstream the *hb-egfa* gene) was replaced with the EGFP-SV40 polyA cassette using Red/ET recombineering technology (Gene Bridges). The 5′ and 3′ homologous arms for recombination were a 50 bp fragment upstream and downstream the first exon of *hb-egfa*, and were included in PCR primers to flank the EGFP-SV40 polyA cassette. We used the same technology to insert a I-SceI site in the final BAC construct, which was purified with Nucleobond BAC 100 kit (Clontech) and co-injected with I-SceI into one-cell-stage zebrafish embryos. A stable transgenic line showing EGFP fluorescence was selected. The allele designation for this line is *pd360*.

#### Generation of *hb-egfb*^*EGFP*^ zebrafish

Transgenic fish were generated using TALEN-directed knock-in^68^ and PhiC31 mediated recombination^69^. Briefly, a pair of obligated heterodimeric TALENs targeting *hbegfb* ATG region (5′-TCAGTCAGACCGACTA-3′ and 5′-TTTCTTTGGGATAGTCCAA-3′) were assembled with standard golden gate assembly^70,71^ and in vitro transcribed with mMESSAGE mMACHINE SP6 Kit (Life Technologies). A 5′ phosphorylated single stranded oligo (ssOligo) was synthesized from Integrated DNA Technologies (IDT) and used as a homology-directed repair (HDR) template (5′- CCTTTTCTTTGGGATAGTCCAAGACACCCCCAACTGAGAGAACTCAAAGGTTACCCCAGTTGGGGTCTGACTGAACCTCCCTGCCTCCAGCGCCGTC-3′). *hbegfb* TALEN mRNAs and ssOligo were co-injected into one-cell stage zebrafish embryos and stable lines were screened by PCR. Next, stable *hbegfb*^*attP*^ F1 zebrafish were sequenced and inter crossed and F2 embryos were injected with PhiC31o mRNA, FLPase mRNA as well as a donor plasmid pERBF-EGFP containing attB, GFP-SV40 polyA, and two FRT sites flanking the vector sequences. Finally, stable *hb-egfb*^*EGFP*^ zebrafish were isolated based on EGFP expression and sequenced to ensure correct integration. The allele designation for this line is *pd361*.

#### Generation of *hb-egfa*KO and *hb-egfb*KO zebrafish

*hb-egfa* and *hb-egfb* mutants were generated using the CRISPR/Cas9 technology. The target sequences were 5′-TGGCCACGTTCATATTTAAGCGG-3′ and 5′-AGCCCTTGCTGTGGTAGCTGTGG-3′ for *hb-egfa* and 5′-CCACCAAACCCAAACATCCGTCG-3′ and 5′- CCACAGCGCTGGCGGTCATAGCA-3′ for *hb-egfb* and led to deletion of a 1512 bp and 2375 bp fragment, respectively. Injected embryos were raised to adulthood and screened using the following primers:

*hb-egfa*KO: Fw1 5′-GCAGGTAACCATACCAGGGATAAAAGG-3′

Rev1 5′-GGTAAAGACGAAAAGACGCAAGACTG-3′

Rev2 5′-CAGGAGGAGGCCAATGATGG-3′

*hb-egfb*KO: Fw1 5′-GCACTGACATCACTCTTGCTCAAC-3′

Rev1 5′-CCATGAATGCAGAAATCTTTGTATTCCTCC-3′

Rev2 5′-GCCTCGAGTTTGACCTTTTCTTCG-3′

The allele designations for these lines are *pd362* and *pd363*.

#### Generation of *hsp70:hb-egfa-P2A-TBFP* zebrafish

*hb-egfa* cDNA was amplified from the embryonic cDNA using the following primers: *hb-egfa*_Fw 5′-ATGAACTTTTTAACAGTCTT-3′ and *hb-egfa*_Rev 5′ CAGAGAGAAATCGTGACATC-3′. Primers were linked to homology arms for Gibson assembly and inserted into an *hsp70-2A-TBFP* vector using Gateway LR Clonase II Enzyme mix (Thermo Fisher Cat#11791020). The final plasmid was co-injected into one-cell stage wild-type embryos with I-SceI. Multiple founders were isolated, propagated, and screened for germline transmission of the transgene selecting for TBFP expression after heat shock. A single line was chosen for maintenance. The allele designation for this line is *pd364*.

#### Generation of *ncoa4EN-cfos:EGFP*, *prp38fbEN-cfos:EGFP*, *ssuh2.4EN-cfos:EGFP*, *hb-egfaEN-cfos:EGFP* and *cfos:EGFP* zebrafish

Putative enhancer regions were PCR-amplified from genomic DNA of 3 dpf EK zebrafish embryos with the following primers: *ncoa4EN:* Fw: 5′-TGTCCCTGGATACACACATACTC-3′, Rev: 5′-CAAAATCAAGCCGTGCCCAA-3′; *prp38fbEN:* Fw: 5′-AATGCCGTCCAAAGCAAGAC-3′, Rev: 5′-GCCATTATGGGGCAACGAGA-3′;

*ssuh2.4EN:* Fw: 5′-TCAGAATAGCTGAAACAATCCTACA-3′, Rev: 5′-GGGAGGTGGTTTGCCATACA-3′; *hb-egfaEN*: Fw: 5′-ACACGTTTCCTCTAGTCCCAG-3′ and Rev: 5′-GGGTTTTACTGTGCTCAAATTGC-3′. The amplified sequences were inserted into pCR8-GW-topoTA (Invitrogen K2500-20) to generate pEntry vectors that were subsequently recombined with PMP6, a Gateway vector containing LR recombination sites upstream of the 95 bp minimal mouse *cfos* promoter driving EGFP^72^. Constructs were injected into fertilized zebrafish embryos along with I-SceI, using standard transgenesis techniques. F1 embryos were genotyped to check for transgene insertion and transmission with the following primers EGFP Fw 5′-ATGGTGAGCAAGGGCGAG-3′, EGFP Rev 5′-CTTGTACAGCTCGTCCATGC-3′. Three stable lines were established. Animals PCR-positive for the transgene were used for experiments. The allele designation for this lines are the following: *hb-egfaEN-cfos:EGFP*, *pd365*, lines 1–3; *prp38fbEN-cfos:EGFP, pd366*, lines 1–2*; ssuh2.4EN-cfos:EGF, pd367*, lines 1–2*; ncoa4EN-cfos:EGFP, pd368*, lines 1–5. As negative control, we generated control lines only carrying a *cfos:EGFP* construct, with an allele designation of *pd369*.

#### Generation of *hb-egfaEN* mutant zebrafish

*hb-egfaEN* was deleted using a pair of gRNAs and the CRISPR/Cas9 technology. gRNAs were designed using the CHOPCHOP website, target sequences were 5′-GTGACGTTTTGCGCCGAGCCGGG-3′ and 5′-TTTGGCATTTCTTCCAGGCATGG-3′, with PAM sequences underlined. To generate knockout animals, gRNAs were co-injected with Cas9 protein (CP01-200, PNA Bio) into one-cell stage embryos. *hb-egfaEN* mutants were screened by PCR with primers *hb-egfEN*KO Fw: 5′- TGTGGGATCATTTGCTTTATCA-3′ and *hb-egfEN*KO Rev: 5′- GCCGCAGCGCACATTACTTTC-3′. Deletions were confirmed by Sanger sequencing (Genewiz, Inc.). The allele designation for this line is *pd371*, lines 1–2.

#### Generation of *ctgfa:mCherry-P2A-NTR* zebrafish

The 5.5 Kb region upstream of the *ctgfa* coding sequence was amplified with the following primers: *ctgfa* Fw: 5′-ATCGATTTTGGCTACTTTCAGCTAAGACTGG-3′ and *ctgfa* Rev: 5′- ATCGATTCTTTAAAGTTTGTAGCAAAAAGAAA-3′. The amplified sequence was inserted into pCR8-GW-topoTA (Invitrogen K2500-20) to generate a pEntry vector that was subsequently recombined with a Gateway vector containing LR recombination sites upstream of a mCherry-P2A-NTR2.0 cassette. The final construct was injected into fertilized zebrafish embryos along with I-SceI, using standard transgenesis techniques. The allele designation for this line is *pd372*.

### Spinal cord injuries in zebrafish

For adult spinal cord injuries, zebrafish were anesthetized using 0.75% 2-phenoxyethanol in fish water. Fine scissors were used to make a small incision through skin and muscle and expose the vertebral column. Then, for transection injuries, the vertebral column was transected halfway between the dorsal fin and the operculum. Complete transection was visually confirmed at the time of surgery. For crush injuries, the spinal cord was crushed using Dumont #5 forceps (Fine Science Tools, 11251-10). Zebrafish were allowed to recover at 28 °C. Sham-injured fish underwent an incision through skin and muscle to expose the spinal cord, which was left intact.

For larval spinal cord injuries, larvae at 3 dpf were anesthetized with 0.02% aminobenzoic-acid-ethyl methyl-ester (MS222, Sigma). Larvae were then transferred into a petri dish coated with agarose. Larvae were placed in a lateral position, and a 30½ G needle was used to perform a dorsal incision and transect the spinal cord. Larvae were allowed to recover at 28 °C^73^.

### RNA- and ATAC-sequencing

All tissue samples were generated from adult zebrafish spinal cord regions collected 2 mm rostral and 2 mm caudal to the lesion site. Sham-operated clutchmate animals were used as controls, collecting the same portion of spinal cord as in injured experimental animals. All samples were prepared in triplicate for each time point.

For ATAC-seq, spinal cords from 60 male and female zebrafish at equal ratios were digested into a single-cell suspension using 0.25% trypsin-EDTA; dissociated cells were processed for FACS sorting using an Astrios sorter, to collect live and single cells. 50,000 cells were processed for ATAC-seq library preparation^61^, and sequencing was performed at the Duke Center for Genomic and Computational Biology on the Illumina HiSeq 4000 platform, with over 40 million 150 bp paired-end reads obtained for each library. Sequences were aligned to the zebrafish genome (danRer10) using Bowtie2 v 2.2.5^74^. The mapped reads were filtered by samtools (v 1.3.1, with parameter -q 30)^75^ and duplicates were removed by picard (v 1.91) Peak calls were determined using MACS2 (v 2.1.0, with parameter -f BAM -g 1.5e9 -q 0.05 --nomodel --shift 37 --extsize 73)^76^, and csaw (v 1.20.0, cutoff *p* value < 0.05)^77^. DiffBind (v 2.14.0) was used to call differential accessible sites. Filter conditions were *p* value < 0.05 and fold change >1.2.

For RNA-seq, RNA was extracted using TRI reagent (Sigma), and genomic DNA was eliminated using the RNA Clean & Concentrator Kit (Zymo Research, R1013). Library preparation and sequencing was performed at the Duke Center for Genomic and Computational Biology using an Illumina HiSeq 4000, with over 40 million 50-bp single-end reads obtained for each library. As for ATAC-seq, reads were aligned to the zebrafish genome (danRer10) using Tophat2 (v 2.1.1)^78^. The mapped reads were filtered by samtools (v 1.3.1, with parameter -q 30^75^ and counted by htseq-count (v 0.6.0)^79^. Differential analyses were performed by Bioconductor package DESeq2 (v1.26.0)^80^.

ATAC-Seq peaks were paired to RNA-Seq differential expression data by annotated nearest gene symbols by ChIPpeakAnno (v3.20.1)^81^. A conservation test was performed using the DNA sequence alignment visualization online tool mVista^82^ and a circle plot was generated using circus (v 0.69-8)^83^. The motif enrichment analysis shown in Supplementary Fig. 6i were performed by AME (Version 5.5.0) at https://meme-suite.org/meme for the enhancer sequence extracted from danRer10: chr14:6760805-6761115. Analyses of predicted Sox2 binding sites in the promoter region of *hb-egfa* shown in Supplementary Fig. 6j were performed using motifmatchr (v 1.12.0)^84^ with motifs downloaded from JASPAR (v2018)^85^. The tracks and heatmaps of ATAC-seq and RNA-seq signals were plotted by Bioconductor package trackViewer (v 1.22.1)^86^ and ComplexHeatmap (v 2.2.0)^87^.

### Single-cell RNA sequencing

Tissue samples were generated from adult zebrafish spinal cord regions collected 2 mm rostral and 2 mm caudal to the lesion site. Sham-operated clutchmate animals were used as controls, collecting the same portion of spinal cord as in injured experimental animals. Samples were processed following the 10X Chromium platform following the manufacturer’s guidelines, using 10X Single-Cell 3′ v3 chemistry (10X Genomics, Pleasanton, USA). For each genotype, injured and sham-injured conditions were prepared and processed in parallel. Sequencing was performed at BGI Genomics using the DNBSEQ platform. The raw reads were processed using the 10x Genomics Cell Ranger pipeline (v.6.0.1). The “cellranger count” was used to identify cell barcodes and feature counts to the fish transcriptomes (danRer11). Libraries were filtered by 200 <number of features <6000 and the percentage of mitochondria genes lower than 20%. The libraries were anchored and integrated using the top 3000 variable features per library in Seurat package (v4.1.1). The “SCT” normalization and reduction on these 3000 features between the libraries was calculated, and the first 30 dimensions used as input for anchoring. Post anchoring, PCA was performed and the first 30 PC’s were used for UMAP dimensionality reduction and subsequent clustering using resolution 0.5. Marker genes per cluster were calculated using Seurat’s “FindAllMarkers” function with parameters (assay = “SCT”, do.print = TRUE, logfc.threshold = 0.5, return.thresh = 0.1, min.pct = 0.5, only.pos = TRUE). Inferences of cell-cell communication were analyzed by CellChat (v 1.4.0) with “CellChatDB.zebrafish” database. Cell types were defined by R scripts “ScType” (v 1.0) by mapping the zebrafish homologs of “Brain” tissue for the scaled data.

### Gene ontology and pathway analyses

Gene ontology analyses were performed using Metascape (v3.5.20230501)^88^. *D. rerio* was selected as species for input and analyses. Pathway and process enrichment analysis was performed using the following ontology sources: GO Biological Processes, KEGG Pathway, Reactome Gene Sets and WikiPathways. All statistically significant ontology terms (*p* value < 0.01) containing at least 3 genes of the initial gene list and with an enrichment factor (ratio between the observed genes in each ontology term and the genes expected by chance) >1.5 were collected and grouped into clusters based on their membership similarities. For each cluster, a subset of enriched ontology terms with a similarity >0.3 used generate networks, where each circle node represents an enriched ontology term. The most significant ontology term in each cluster based on *p* values was used to represent the cluster and is shown in the label. Pathway analysis was performed using Ingenuity Pathway Analysis (IPA, v. 70750971, QIAGEN Inc., Redwood City, CA, USA)^89^ and Panther (v17.0)^42,90^.

### RNA isolation and qRT-PCR

Spinal cords were homogenized in Trizol, and RNA was extracted using the standard Trizol protocol. Genomic DNA removed using RNA clean and Concentrator Kit (Zymo Research, R1013). cDNA synthesis was performed using Transcriptor First Strand cDNA Synthesis Kit (Roche, 04897030001) and qPCR run was performed with LightCycler 480 SYBR Green I Master (Roche, 04707516001), and with LightCycler 480 software 1.5.0 SP4. All gene expression values were normalized to beta-actin levels. Primers used were the following:

*hb-egfa*: Fw 5′-GGGCCCTCATGCATATGTGA-3′, Rev 5′-AGAATTTCCACACGGTCGCC-3′;

*hb-egfb*: Fw 5′-AGCGGGATTTACGCACTCAT-3′, Rev 5′-GCATGCAGAATATCTTAATGCCA-3′;

neurod4 Fw 5′-CGTCCATCCATCCAAGGGAG-3′, Rev 5′- CGTCCATCCATCCAAGGGAG-3′;

sox11b Fw 5′-CCTTTGCCTCTCTCTGGTGG-3′, Rev 5′ CAGGAGTGCAATAGTCCGGG-3′;

adcyap1b Fw 5′-ATCCGCCAGAGAAAAGAACGG-3′, Rev 5′ CGCGAACTGCCATCAGAGA-3′;

*sox2:* Fw 5′-GGGCACGGGGAACACCAACT-3′, Rev 5′- TGGTCGCTTCTCGCTCTCGG-3′;

*actin*: Fw 5′-GACAACGGCTCCGGTATG-3′, Rev 5′-CATGCCAACCATCACTCC-3′.

### EdU, biocytin and human recombinant (HR) HB-EGF treatment

For EdU incorporation, zebrafish were injected intraperitoneally with 10 μl of 10 mM 5-ethynyl-2′-deoxyuridine (EdU, Molecular Probes, A10055), and tissue was collected 24 h after a single treatment, or after daily treatments for 1 or 2 weeks. For biocytin treatment used for anterograde axon tracing, adult fish were anaesthetized using 0.75% 2-phenoxyethanol in fish water. Scissors were used to make a small incision on the dorsal side of the skin and to transect the spinal cord 2 mm rostral to the original spinal cord transection site. A biocytin-soaked gelfoam gelatin sponge was applied at a new injury site (Gelfoam, Pfizer, 09-0315-08; Alexa Fluor 594 Biocytin, Thermo Fisher Scientific A12922). The incision was closed and glued using Vetbond tissue adhesive material (Santa Cruz Biotechnology, sc-361931). Tissue was collected 24 hrs after biocytin application.

For HR-HB-EGF treatment, HR-HB-EGF protein (R&D Systems, 259-HE-250) was conjugated with hydrogel (see below) and 3 μl of solution was locally injected just rostral to the original spinal cord transection site with a Hamilton syringe.

### Synthesis of hyaluronic acid-based hydrogel

Hyaluronic acid (HA) was reacted with ureidopyrimidinone (UPy) moiety following previously published protocol to obtain Upy conjugated HA (HA-Upy)^43^. Next, HA-Upy was oxidized by sodium periodate to obtain dialdehyde containing HA-Upy (HA-Upy-DA). Briefly, HA-Upy was dissolved in deionized water at 5 mg/ml. Sodium periodate (NaIO_4_, one equivalent with respect to the sugar ring of HA-Upy), dissolved in 5 ml of water, was added into the HA-Upy solution. After stirring for about 2 h at room temperature, the reaction mixture was quenched with excess of ethylene glycol (10 equivalent with respect to NaIO_4_) for about 30 min. The mixture was purified via dialysis against DI water. The solution was then freeze-dried to obtain HA-Upy-DA. The aldehyde content in HA-Upy-DA was determined by ^1^HNMR following reaction of tert-butyl carbazate (t-BC) with HA-Upy-DA, and the degree of oxidation was calculated by comparing the signal of t-BC group (1.38 ppm, 9H) to that of −NHCOCH_3_ methyl group of HA (1.9 ppm, 3H)^91^. The aldehyde content was found to be 9 ± 1%. Fluorescently labeled HA-Upy-DA was synthesis similarly by conjugating fluorescein isothiocyanate (FITC) to HA-Upy followed by oxidation of FITC conjugated HA-Upy. Hydrogels were prepared by dissolving the polymers in 1X PBS (pH 7.4) containing 0.1% bovine serum albumin (BSA). To encapsulate growth factor into the hydrogel, the growth factor (Recombinant Human HB-EGF Protein, R&D Systems) was first dissolved in PBS containing 0.1% BSA at 50 ug/100 μl. The solution of the growth factor was then added to freeze-dried HA-Upy-DA (3.5 wt%). Growth factor loaded hydrogel was obtained upon complete dissolution of the HA-Upy-DA. The hydrogel was transferred to a Hamilton syringe for application.

### Histological analysis in zebrafish

PFA-fixed tissue was rinsed in phosphate buffer, then cryoprotected in 30% sucrose. Samples were embedded in optimal cutting temperature compound (OCT) (Tissue-Tek) and frozen in a dry ice. Twenty μm longitudinal (horizontal) or 16 μm transversal cryosections were used for histology. For immunohistochemistry, tissue sections were rehydrated in PBS, permeabilized in PBS containing 0.2% Triton X-100, and incubated with blocking buffer (5% goat serum in PBS-Tween) for 1 h at room temperature. Sections were incubated overnight with primary antibodies diluted in blocking agent, washed in PBS, and treated for 1 h in secondary antibodies and DAPI (Thermo Fisher Scientific, D3571, 1:5000). Following washes, sections were mounted in in VECTASHIELD® Antifade Mounting Medium (Vector Laboratories H-1000-10). Primary antibodies used for adult fish experiments were: rabbit anti-GFP (Life Technologies, A11122, 1:500), chicken anti-GFP (Aves Labs, GFP-1020, 1:500), mouse anti-GFAP (ZIRC, Zrf1, 1:200), rabbit anti-GFAP (Sigma, G9269, 1:200), rabbit anti-Sox2 (Abcam, ab97959, 1:200), mouse anti-HuC/D (Invitrogen, A-21271, 1:100), mouse anti-acetylated-α-tubulin (Sigma, T6793, 1:1000), rabbit anti-dsRed (Clontech, 632496, 1:200), mouse anti-PCNA (Sigma, P8825, 1:200). Secondary antibodies (Life Technologies, 1:200) used in this study were highly cross-absorbed Alexa Fluor 488/633/594 goat anti-rabbit, anti-mouse or anti-chicken antibodies (Alexa Fluor 488 Goat anti-Rabbit, Thermo Fisher Scientific, A-11034; Alexa Fluor 594 Goat anti-Rabbit, Thermo Fisher Scientific, A-11037; Alexa Fluor 488 Goat anti-Mouse, Thermo Fisher Scientific, A-11029; Alexa Fluor 633 Goat anti-Mouse, Thermo Fisher Scientific, A-21050; Alexa Fluor 594 Goat anti-Mouse, Thermo Fisher Scientific, A-11032; Alexa Fluor 488 Goat anti-Chicken, Thermo Fisher Scientific, A-11039; Alexa Fluor 633 Goat anti-Mouse, Thermo Scientific, A-21052). EdU staining was performed using 20 μM Alexa Fluor 594 azide (Molecular Probes, A10270). All confocal images were acquired with either a Zeiss LSM 700 or LSM 880 microscope with Zen 2010 B SP1.

In situ hybridization was performed on cryosections of paraformaldehyde-fixed spinal cord^19^, using an Intavis in situ robot. To generate probes, target sequences were placed upstream of a T7 promoter and gBlock fragments were ordered at IDT. Probes were generated using T7 RNA polymerase (M0251, New England BioLab). Signals were visualized by immunoassay using an anti-DIG-AP (alkaline phosphatase) antibody (11093274910, Sigma-Aldrich) and subsequent catalytic color reaction with NBT (nitroblue tetrazolium) (11383213001, Sigma-Aldrich)/BCIP (5-bromo-4-chloro-3-indolyl-phosphate) (11383221001, Sigma-Aldrich). Sections were imaged using a Leica DM6000 compound microscope with Leica Application Suite X (v.3.4.2). Whole mount immunofluorescence was performed using established protocols^92^. Larvae were imaged using a Zeiss Axio Zoom microscope with Zen Pro 2012.

### Swim capacity assays

Swim capacity was measured by exercising fish in groups of 8–12 in a 5L swim tunnel respirometer device (Loligo, SW100605L, 120 V/60 Hz)^19^. Fish were acclimated for 20 min at a fixed low water current in the enclosed tunnel, then water current velocity was increased every 2 min and fish swam against the current until they reached exhaustion. For each animal reaching exhaustion, swim time and current velocity at exhaustion were recorded.

### Zebrafish larvae experiments

For imaging experiments, 3 days post fertilization (dpf) larvae were placed in a 50 ml falcon tube in egg water and immersed in a 38 °C water bath for 1 h. Larvae were then placed in a petri dish in a 28 °C incubator. At 6 h post heat shock, larvae were imaged using a Zeiss Axio Zoom microscope, with settings unchanged between experimental and control groups.

For qPCR analyses, 3 dpf larvae were placed in a 50 ml falcon tube in egg water and immersed in a 38 °C water bath for 1 h. Larvae were then placed in a petri dish in a 28 °C incubator. At 3 h post heat shock, single larvae were lysed in Trizol. DNA and RNA were extracted using the manufacturer instructions for genotyping and qPCR analyses, respectively.

### Mice

All experimental procedures with mice were performed in compliance with animal protocols reviewed and approved by the Duke IACUC. Wild-type male and female C57BL/6 mice were purchased from The Jackson Laboratory and used for all experiments. For neonatal experiments, injuries were performed at postnatal day 3 (P3). For experiments with adults, 3 month-old mice were used. Mice were housed in a temperature-controlled (~18–23 °C, 40–60% humidity) and enriched environment, with a 12 h light/dark cycle, and provided standard chow and water.

### Spinal cord injuries in mice

For neonatal spinal cord crush injury, mice at postnatal day 3 (P3) were anaesthetized by hypothermia on an ice bed. A laminectomy was performed at thoracic level (T9-T10) to completely expose the spinal cord. The spinal cord was crushed for 2 sec using forceps. After visually confirming establishment of the injury, muscle and skin were sutured in layers with 6-0 absorbable sutures. Mice were warmed until awake and placed into a cage containing bedding from their original cage for at least 30 min before the mother was returned. In case of bladder dysfunction, bladder expression was performed daily. Sham-operated pups underwent the same procedure involving laminectomy without spinal cord crush^3^.

For adult crush and transection injuries, mice were anesthetized with ketamine (50–100 mg/kg) and xylazine (5–10 mg/kg) and given antibiotics (gentocin, 1 mg/kg)^3,93^. A midline incision was made over the thoracic vertebrae, followed by removal of the T9-T10 lamina to expose the spinal cord. The spinal cord was fully crushed for 2 s with Dumont #5 forceps (Fine Science Tools, 11251-10) modified to 0.5 mm tip^94^. For transection injuries, the spinal cord was cut using iridectomy scissors. Muscles were then sealed with 6-0 absorbable sutures and the skin was closed with wound clips. Two ml of sterile saline was administered subcutaneously (sq) to prevent de-hydration. During recovery, mice were kept on a heating pad and received antibiotic agents (1 mg/kg gentocin) and saline daily for 5 days. Manual bladder expression was performed twice per day until tissue harvest. Sham-operated mice underwent laminectomy without spinal cord crush and received all post-operative cares as injured mice.

### Histological analyses in mice

Mice were given a lethal dose of anesthesia and were transcardially perfused with PBS followed by 4% paraformaldehyde (PFA). PFA-fixed tissues were post-fixed in 4% paraformaldehyde, rinsed in phosphate buffer, then cryoprotected in 30% sucrose. Samples were embedded in OCTand frozen in dry ice. Longitudinal horizontal sections were cut on a cryostat at 20-μm thickness and stored at −20 °C until processed. Before staining, sections were warmed to room temperature, permeabilized using Triton X-100, treated with blocking agent and incubated over night at 4 °C with primary antibodies. Following washes, sections were incubated washed in PBS and mounted in VECTASHIELD(R) Antifade Mounting Medium (Vector Laboratories H-1000-10). Primary antibodies used for mouse immunofluorescence anti-EGFP (rabbit, A11122, Life Technologies, 1:500), anti-EGFP (chicken, GFP-1020, Aves Labs, 1:500), mouse anti-GFAP (Sigma, G3893, 1:500), rabbit anti-Sox2 (Abcam, ab97959, 1:200), mouse anti-HuC/D (Invitrogen, A-21271, 1:100), rabbit anti-5-HT (Immunostar, 20079, 1: 5000), rabbit anti Ki67 (Abcam, ab15580, 1:200), rabbit anti-fibronectin (Sigma, F3648, 1:200), rat anti-CD68 (Bio-Rad, MCA1957, 1:600), F4/80 (Biorad, MCA497R, 1:200). Secondary antibodies (Life Technologies, 1:200) used in this study were highly cross-absorbed Alexa Fluor 488/633/594 donkey or goat anti-rabbit, anti-mouse, anti-rat or anti-chicken antibodies (Alexa Fluor 488 Goat anti-Rabbit, Thermo Fisher Scientific, A-11034; Alexa Fluor 594 Goat anti-Rabbit, Thermo Fisher Scientific, A-11037; Alexa Fluor 488 Goat anti-Mouse, Thermo Fisher Scientific, A-11029; Alexa Fluor 633 Goat anti-Mouse, Thermo Fisher Scientific, A-21050; Alexa Fluor 594 Goat anti-Mouse, Thermo Fisher Scientific, A-11032; Alexa Fluor 488 Goat anti-Chicken, Thermo Fisher Scientific, A-11039; Alexa Fluor 633 Goat anti-Mouse, Thermo Scientific, A-21052; Alexa Fluor 633 Goat anti-Rabbit, Thermo Scientific, A-21071; Alexa Fluor 568 Goat anti-Rat, Thermo Scientific, A-11007; Alexa Fluor 594 Rabbit anti-Goat, Thermo Scientific, A11080; Alexa Fluor 488 Donkey anti-Goat, Thermo Fisher Scientific, A-11055; Alexa Fluor 594 Donkey anti-Goat, Thermo Fisher Scientific, A-11058). Confocal images were acquired with Zeiss LSM 700 or Zeiss LSM 880 microscopes.

### Virus production and titers

A triple-plasmid transfection protocol was used to produce recombinant AAV vectors in suspension HEK293s (obtained from the University of North Carolina Vector Core). Specifically, the transfected plasmids include a capsid-specific helper plasmid (AAV.cc47, containing AAV2 Rep and AAV9 variant Cap genes^53^), the adenoviral helper plasmid pXX680, and pTR-Enhancer-HSP68-GFP plasmids (encoding different enhancer elements), flanked by inverted terminal repeats (TRs) derived from the AAV2 genome. Culture media was harvested 6 days post transfection and cells were pelleted via centrifugation (1000 × *g* × 15 min) and discarded. Viral particles were precipitated from the culture media overnight at 4 °C with polyethylene glycol (PEG; final concentration of 12%). Media was subsequently centrifuged at 3000 × *g* × 1 h and discarded. The PEG pellet was resuspended in formulation buffer (1x PBS with 1 mM MgCl_2_ and 0.001% puronic F-68) and treated with DNase at 37 °C for 1 h. Viral vectors were purified using iodixanol density gradient ultracentrifugation. Vectors were subsequently subjected to buffer exchange using Pierce Protein PES centrifugation columns (100,000 MWCO, Thermo Scientific, catalog no. 88524). Following purification, viral genome titers were determined via quantitative PCR using a Roche Lightcycler 480 (Roche Applied Sciences). Quantitative PCR primers were designed to specifically recognize the AAV2 inverted terminal repeats (forward, 5′-AACATGCTACGCAGAGAGGGAGTGG-3′; reverse, 5′-CATGAGACAAGGAACCCCTAGTGATGGAG-3′) (Integrated DNA Technologies). 10^11^ virus particles were injected into neonatal mice, and 10^12^ into adult mice, by temporal vein and tail vein injection, respectively.

### Data analysis and statistics

The number of animal analyzed for all quantifications (*n*) is indicated in the figure panels. The number of trials for each experiment (*N*) is indicated in Figure legends.

Quantification of tissue bridging was performed on Z-stacks, while quantification of biocytin and EdU incorporation in Sox2^+^ or HuC/D^+^ cells was performed on single-plane images. Glial bridge diameter was calculated using ImageJ software, measuring 3–5 longitudinal, horizontal sections per fish with the thickest bridge, which was identified by staining for axons and glia (acetylated α-tubulin and GFAP, respectively). Measurements at the regenerating lesion site were normalized on diameter of the spinal cord caudal to the lesion, as performed previously^19^. Biocytin-labeled axons were quantified from transverse sections, counting number of particles (each one representing the cross-section of an axon) using the “threshold” and “particle analysis” tools in the ImageJ software. We analyzed 3–5 transverse sections per fish, which were located ~0.5 mm caudal to the lesion; we then normalized measurements on signals detected ~1 mm rostral to the lesion core for each fish, as done previously^19^. Cycling of Sox2^+^ ERGs was quantified by manually counting the number of Sox2^+^ cells labeled with EdU surrounding the central canal, using transverse sections located ~0.5 mm caudal to the lesion. Labeling of HuC/D^+^ cells was quantified on longitudinal horizontal sections, in 0.3 mm regions rostral and caudal to the lesion site. Three to five longitudinal horizontal sections per animal were analyzed and averaged. qPCR experiments were performed 3 times, 3 technical replicates were run each time. Fold changes were calculated with the 2-ΔΔCT method.

For violin plots, solid lines indicate the group median; dotted lines indicate the 25th and 75th quartiles. All samples are shown in each violin plot. Sample sizes, statistical tests, and *p* values are indicated in the figures or the legends. All statistical analyses were performed using GraphPad Prism 9 software.

For zebrafish experiments, we injured ~ 5–15 fish per group, according to genotype availability after PCR genotyping. After injury, animals were randomly assigned to each experimental group (e.g. HR-HB-EGF treatment/BSA vehicle) and end-point. Viability was ~65%. For neonatal mouse experiments, we injured ~10 mice per group, and viability was ~70%. For adult mouse experiments, we injured 3–5 mice per group, with a viability of ~90%. No animals were excluded from the analyses. Investigators were not blinded during experiments and outcome assessment.

### Reporting summary

Further information on research design is available in the Nature Portfolio Reporting Summary linked to this article.

### Supplementary information

Supplementary Information

Peer Review File

Description of Additional Supplementary Files

Supplementary Data 1

Supplementary Data 2

Supplementary Data 3

Supplementary Data 4

Supplementary Data 5

Supplementary Movie 1

Supplementary Movie 2

Supplementary Movie 3

Supplementary Movie 4

Supplementary Movie 5

Supplementary Movie 6

Supplementary Movie 7

Supplementary Movie 8

Reporting Summary

### Source data

Source Data

## Data Availability

The bulk RNA, ATAC and single-cell RNA sequencing datasets generated in this study have been deposited in the Gene Expression Omnibus (GEO) archive. The accession number for bulk RNA and ATAC sequencing is GSE193503. The accession number for single-cell RNA-seq data is GSE213435. The remaining data are available within the article or Source Data file. Unique biological materials are available from the corresponding author upon request. Source data are provided with this paper.
