# Supplementary material for: Spinal cord repair is modulated by the neurogenic factor Hb-egf under direction of a regeneration-associated enhancer
Source: Nat Commun. 2023 Aug 11;14:4857. doi: 10.1038/s41467-023-40486-5 (PMC10421883; doi:10.1038/s41467-023-40486-5)
Supplement: Supplementary file 1 — Supplementary Information [file 41467_2023_40486_MOESM1_ESM.pdf]

Supplementary Materials

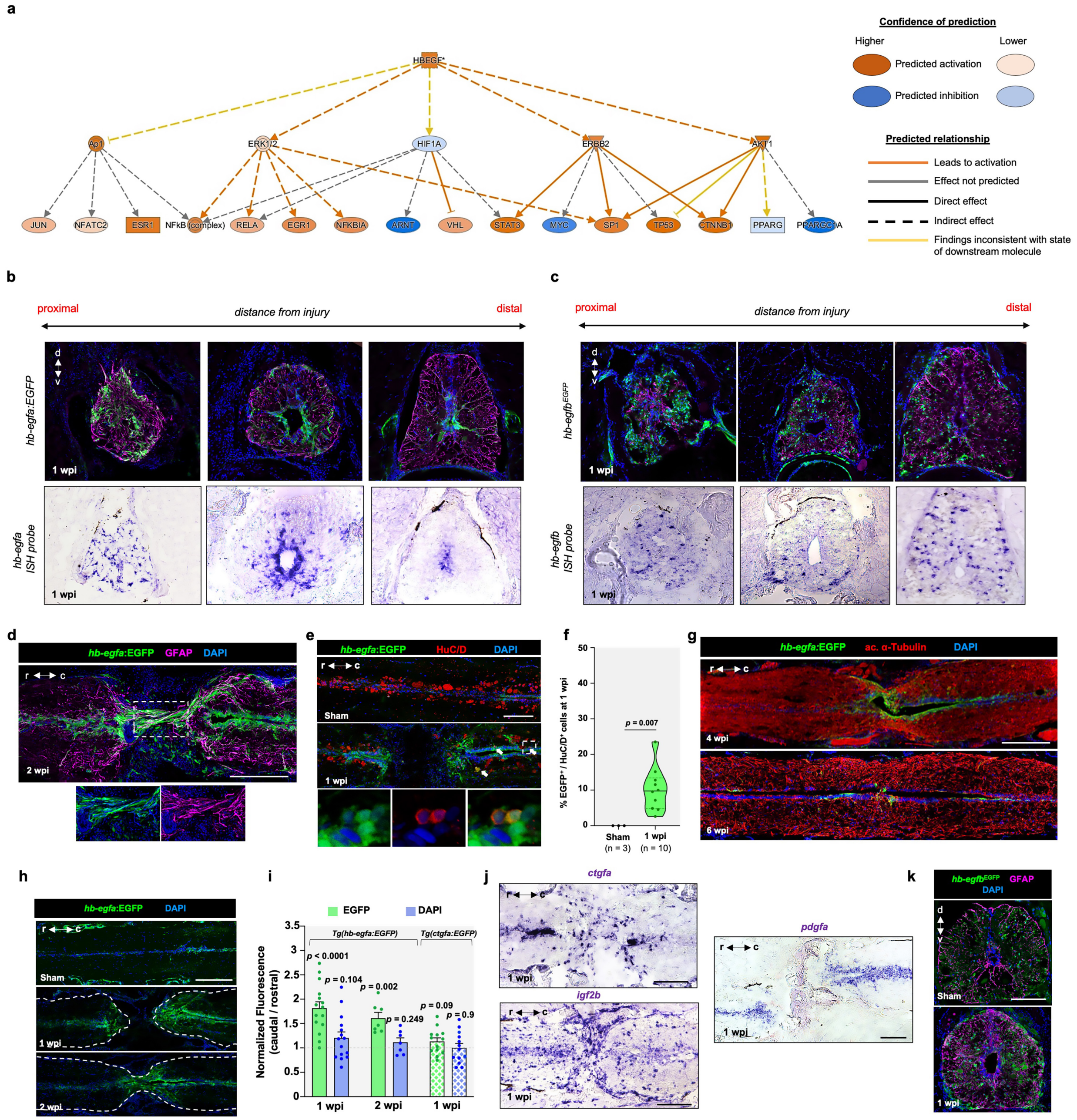

Supplementary Fig. 1. Induction of *hb-egfa* after spinal cord injury.

(a) Mechanistic Hb-egf network from IPA upstream regulator analysis.

(b, c) Transverse sections of adult zebrafish spinal cord showing comparison of *hb-egfa* **(b)** and *hb-egfb* **(c)** -directed EGFP fluorescence (top) and mRNA (bottom) at different distances from the lesion epicenter, at 1 wpi. N = 3.

(d) Expression of *hb-egfa*:EGFP at sites of tissue bridging in adults at 2 wpi. GFAP (magenta) stains glial cells. N = 3.

(e) Longitudinal sections of regenerating spinal cord at 1 wpi, showing cells co-expressing Hb-egfa (green) and the neuronal marker HuC/D. Arrows indicate cells expressing both markers. Quantification shown in **(f)**.

(g) Expression of *hb-egfa*:EGFP at 4 and 6 wpi. Acetylated α-Tubulin stains axons. N=3.

(h) Longitudinal sections of spinal cords from *hbegfa:EGFP* BAC reporter fish at 1 and 2 wpi, indicating differential rostrocaudal distribution of EGFP fluorescence sections.

(i) Quantification of EGFP and DAPI fluorescence in the rostral and caudal sides of spinal cord lesions at 1 and 2 wpi in *hb-egfa:EGFP* fish (filled columns) and at 1 wpi in *ctgfa:egfp* fish (dotted columns). *ctgfa*:EGFP, by contrast, shows no differential distribution in rostral and caudal domains. *hb-egfa:EGFP*: n = 15 and 7 at 1 and 2 wpi, respectively; *ctgfa:EGFP*: n = 13 at 1 wpi. Animals were exhamined over 2 indipendent experiments. A one-sample two-tailed t-test was used for comparison. Error bars indicate SEM.

(j) Longitudinal sections of injured spinal cord at 1 wpi, showing expression of *ctgfa*, *igf2b* and *pdgfa* mRNA by ISH. N = 2.

(k) Transverse section of adult spinal cord showing expression of *hb-egfb*^{EGFP} and the glial marker GFAP in sham-injured tissue and at 1 wpi. N = 3.

Dashed area in **d** and **e** indicate region magnified, and in **h** spinal cord stumps and sites of tissue bridging. Scale bars 100 µm in **e** and **k**, 200 µm in **d**, **g**, **h**, **j**. A two-tailed Mann-Whitney test was used for comparisons in **f** and two-tailed unpaired t-test for comparisons in **i**. “n” in **f**= number of animals used for the experiments. r, rostral, c, caudal, d, dorsal; v, ventral.

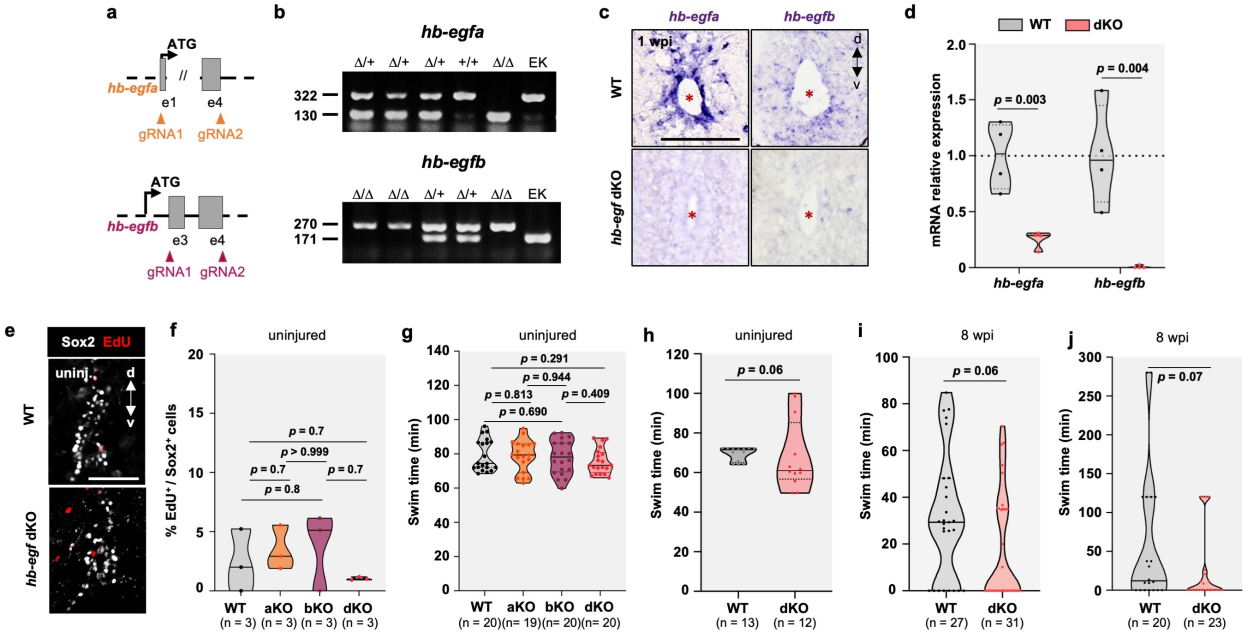

Supplementary Fig. 2. Swim capacity and expression of *hb-egf* paralogues in dKO mutants.

(a, b) Cartoon and genotyping indicating the exonic regions deleted to generate *hb-egfa* and *hb-egfb* mutations.

(c) *In situ* hybridization of transverse sections of spinal cord, showing loss of *hb-egfa* and *hb-egfb* induction at 1 wpi in dKO mutants compared to wild-type (WT) clutchmates. N=2.

(d) Quantitative RT-PCR indicating decreased expression of *hb-egfa* and *hb-egfb* in dKO mutant embryos compared to wild-type controls.

(e, f) Ependymal cell cycling assessed by EdU (red) incorporation in spinal cords of uninjured WT, *hb-egfaKO, or hb-egfbKO* and *hb-egf* dKO fish. Analyses were performed after 1 week of daily Edu injections. Quantification shown in **f**. N = 1.

(g) Swim capacity of uninjured WT, *hb-egfaKO, or hb-egfbKO* and *hb-egf* dKO fish against increasing water currents. The graph shows the time of exhaustion for fish in all groups. N = 2.

(h) Endurance swim testing of uninjured WT and *hb-egf* dKO fish. Fish were swum at a constant current of 35 cm/sec and time before they were exhausted was recorded.

(i) Swim capacity of WT and *hb-egf* dKO fish against increasing water currents. N = 2.

(j) Endurance swim testing of uninjured WT and *hb-egf* dKO fish. Fish were swum at a constant current of 35 cm/sec and time before they were exhausted was recorded.

Two-tailed unpaired t-tests were used for comparisons in **d** and two-tailed Mann-Whitney tests were used for comparisons in **f, g, h, i,** and **j.** Scale bar in **c** 100 µm, 50 µm in **e**. d, dorsal; v, ventral. “n” = number of animals used for the experiments. Source data are provided as a Source Data file.

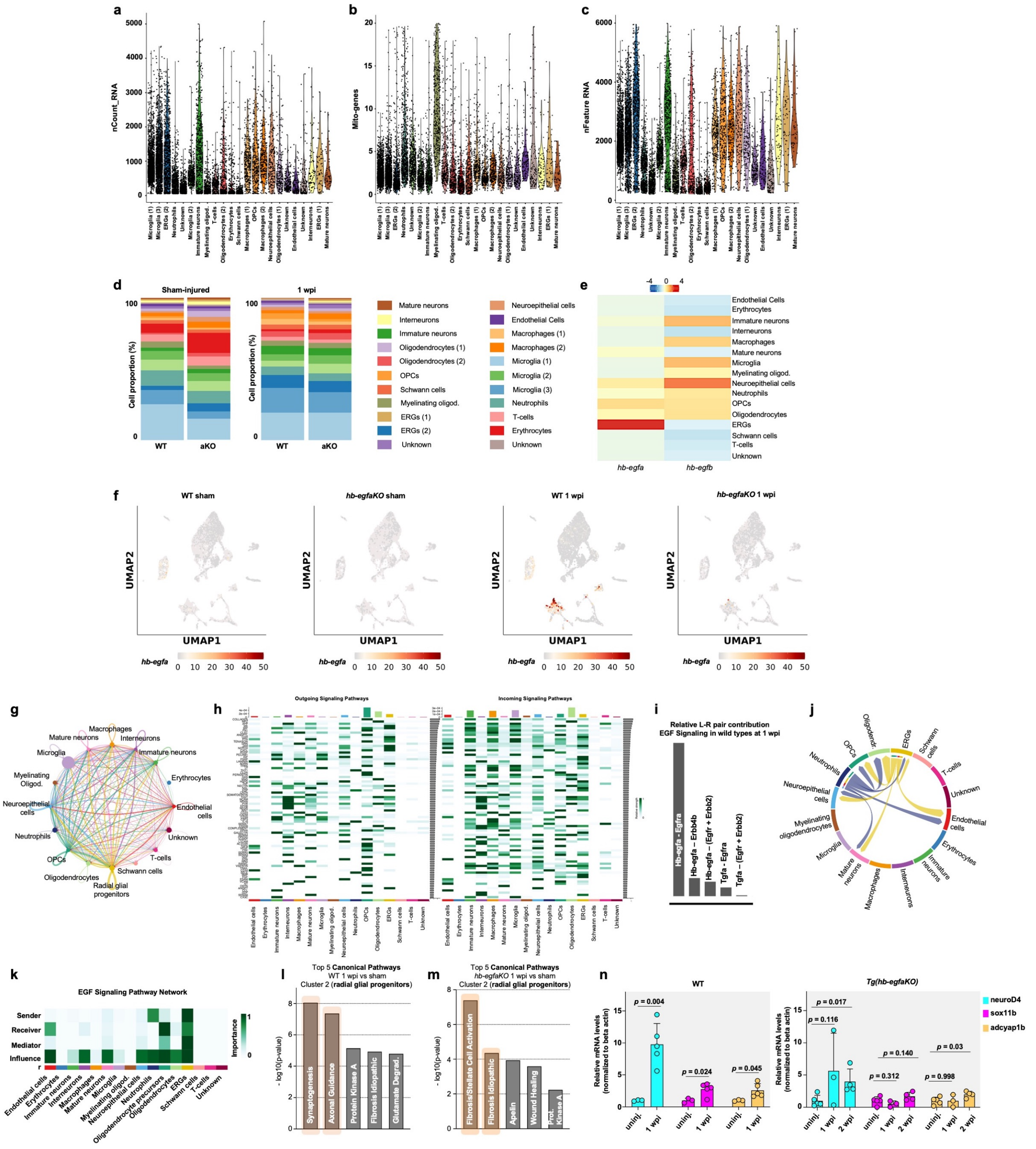

Supplementary Fig. 3. scRNAseq of wild-type and *hb-egfa*KO sham injured and 1 wpi spinal cords.

(a-c) Violin plots showing numbers of RNA counts (nCount_RNA), proportion of mitochondrial RNA (percent.mito), and number of RNA features (nFeature_RNA) for the cell clusters identified by scRNAseq.

(d) Proportion of cells in each cluster identified in sham-injured and 1 wpi wild-type (WT) and *hb-egfa*KO spinal cords.

(e) Heatmap showing expression of *hb-egfa* and *hb-egfb* mRNA by scRNAseq in distinct spinal cord cell populations.

(f) Changes in *hb-egfa* expression in sham-injured and 1 wpi WT and *hb-egfa*KO clusters.

(g) Overview of cell-cell interaction network during spinal cord regeneration. Thickness of lines joining the different cell clusters indicates interaction strength. Bubble size indicates cell contributing to the respective cluster.

(h) Heatmap showing the contribution of all cell clusters identified to the significant signaling networks identified by CellChat analyses. Size of the rectangles at the top of each cell type indicates its overall contribution.

(i) Relative contribution of each ligand-receptor pair to the overall EGF signaling network.

(j) Circle plot and **(k)** heatmap showing the relative contribution of each cell cluster to the EGF signaling network during spinal cord regeneration.

(l, m) Top 5 canonical pathways in radial glial progenitor clusters at 1 wpi in WT **(l)** and *hb-egfa*KO **(m)** spinal cords.

(n) qPCR analysis to assess expression of the neuronal marker genes *neuroD*4, *sox11b* and *adcyap* at 1 and 2 wpi in bulk spinal cord tissue of WT and *hb-egfaKO* zebrafish. *sox11b* and *adcyap1b* expression levels increase at 1 wpi in *hb-egf* WT fish, yet they are unchanged compared to uninjured spinal cords at both 1 and 2 wpi in *hb-egfaKO* fish. Similarly, neuroD4 displays a smaller expression level increase in *hb-egfaKO* fish compared to *hb-egf* WT, at both 1 and 2 wpi. WT n=3 and 5 in uninjured and 1 wpi condition; *Tg(hb-egfaKO)* n=4, 3 and 4 in uninjured, 1 and 2 wpi samples, respectively. A two-tailed unpaired t-test was used for comparisons. Error bars indicate SEM.

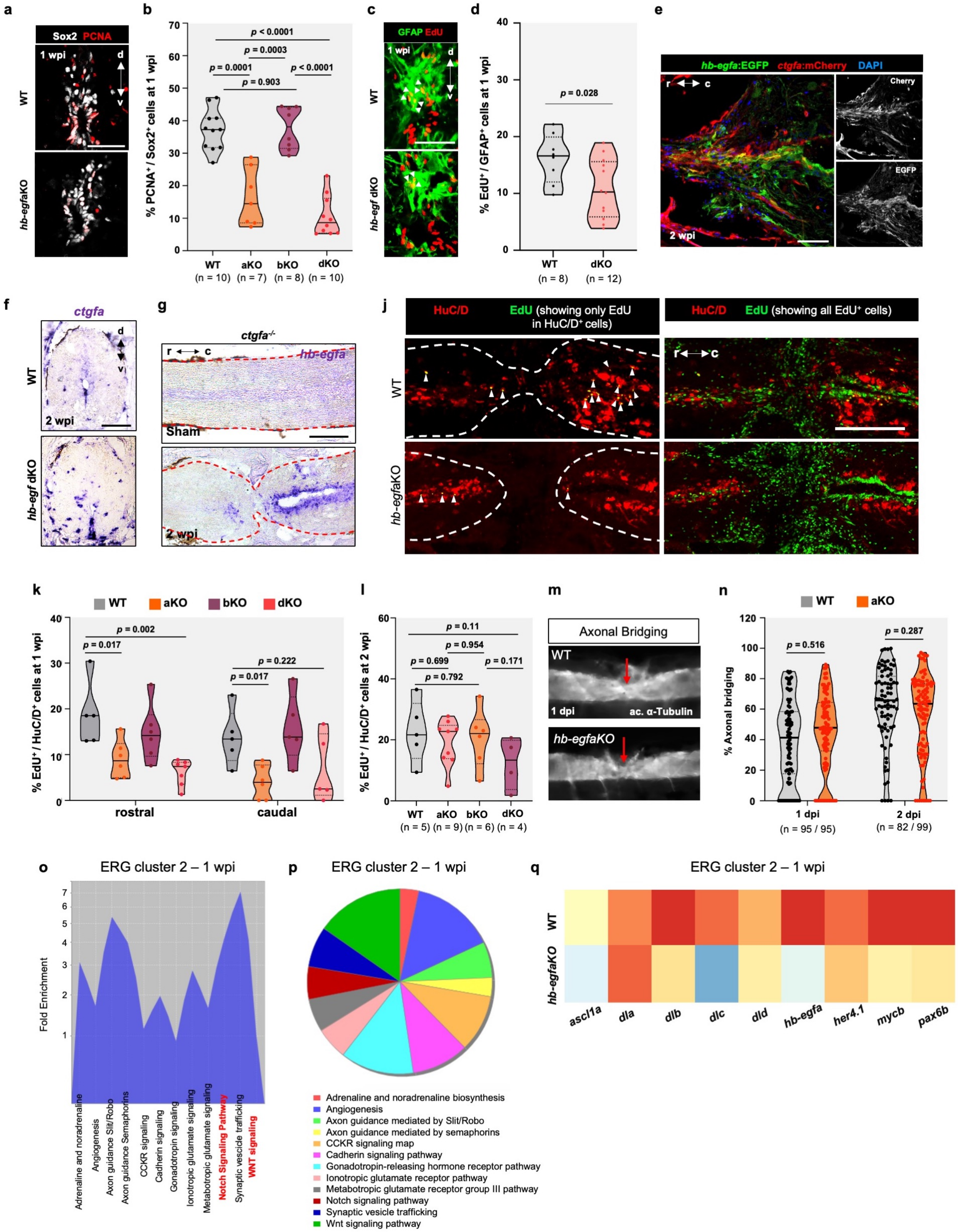

a
Sox2 PCNA
1 wpi
WT
hb-egfaKO
b
% PCNA+ / Sox2+ cells at 1 wpi
p < 0.0001
p = 0.0003
p = 0.0001
p < 0.0001
p = 0.903
WT (n = 10)
aKO (n = 7)
bKO (n = 8)
dKO (n = 10)
c
GFAP EdU
WT
hb-egf dKO
d
% EdU+ / GFAP+ cells at 1 wpi
p = 0.028
WT (n = 8)
dKO (n = 12)
e
hb-egfa:EGFP ctgfa:mCherry DAPI
Cherry
EGFP
2 wpi
f
ctgfa
WT
2 wpi
hb-egf dKO
g
ctgfa-/-
hb-egfa
Sham
2 wpi
j
HuC/D EdU (showing only EdU in HuC/D+ cells)
HuC/D EdU (showing all EdU+ cells)
WT
hb-egfaKO
k
WT aKO bKO dKO
% EdU+ / HuC/D+ cells at 1 wpi
p = 0.002
p = 0.017
p = 0.222
p = 0.017
rostral
caudal
l
% EdU+ / HuC/D+ cells at 2 wpi
p = 0.11
p = 0.954
p = 0.699
p = 0.171
p = 0.792
WT (n = 5)
aKO (n = 9)
bKO (n = 6)
dKO (n = 4)
m
Axonal Bridging
WT
1 dpi
ac. α-Tubulin
hb-egfaKO
n
WT aKO
% Axonal bridging
p = 0.516
p = 0.287
1 dpi (n = 95 / 95)
2 dpi (n = 82 / 99)
o
ERG cluster 2 – 1 wpi
Fold Enrichment
Adrenaline and noradrenaline
Angiogenesis
Axon guidance Slit/Robo
Axon guidance Semaphorins
CCKR signaling
Cadherin signaling
Gonadotropin signaling
Ionotropic glutamate signaling
Metabotropic glutamate signaling
Notch Signaling Pathway
Synaptic vesicle trafficking
WNT signaling
p
ERG cluster 2 – 1 wpi
Adrenaline and noradrenaline biosynthesis
Angiogenesis
Axon guidance mediated by Slit/Robo
Axon guidance mediated by semaphorins
CCKR signaling map
Cadherin signaling pathway
Gonadotropin-releasing hormone receptor pathway
Ionotropic glutamate receptor pathway
Metabotropic glutamate receptor group III pathway
Notch signaling pathway
Synaptic vesicle trafficking
Wnt signaling pathway
q
ERG cluster 2 – 1 wpi
WT
hb-egfaKO
ascl1a
dla
dlb
dlc
dld
hb-egfa
her4.1
mycb
pax6b

Supplementary Fig. 4. Effects on gliogenesis and neurogenesis by Hb-egfa.

(a) Transverse sections of wild-type (WT) and *hb-egfaKO* spinal cords at 1 wpi, stained for ependymal cells ($Sox2^+$, white) and PCNA (red).

(b) Quantification of $PCNA^+Sox2^+$ ependymal cells at 1 wpi in different mutant backgrounds. N = 2.

(c, d) Cycling of GFAP-positive glial cells assessed by EdU (red) incorporation in spinal cords of WT and *hb-egf* dKO fish. N = 2.

(e) Co-localization between *hb-egfa*:EGFP and *ctgfa*-directed mCherry at 2 wpi. N = 2.

(f) Transverse sections of WT and *hb-egf dKO* spinal cords showing expression of *ctgfa* mRNA in ventral domains by ISH at 2 wpi. N=2.

(g) Longitudinal sections of *ctgfa*$^{-/-}$ zebrafish spinal cord showing expression of *hb-egfa* mRNA by ISH at 2 wpi. N = 2.

(j) Longitudinal sections of spinal cord at low magnification, indicating EdU-labeled neurons at 1 wpi in WT and *hb-egfa*KO samples.

(k) Quantification of EdU^+HuC/D^+ cells in different mutant backgrounds at 1 wpi in rostral and caudal domains of a transection injury at 1 wpi. N = 2.

(l) Quantification of EdU^+HuC/D^+ cells in different mutant backgrounds at 2 wpi after transection injury. N = 2.

(m) Whole mount images of WT and *hb-egfaKO* larval spinal cord at 1 day post injury (dpi) stained for the axonal marker acetylated α-tubulin. Quantification of axonal bridge at 1 and 2 dpi in **(n)**.

(o, p) Bioinformatic comparison of ERG clusters of WT and *hb-egfaKO* fish at 1 wpi. Shown are the signaling pathways enriched in lists of gene differentially modulated in the two conditions. Notch and Wnt signaling pathways are among overrepresented pathways.

(q) Heat maps showing level of expression of Wnt and Notch pathway components at 1 wpi in ERG clusters of WT and *hb-egfaKO* fish.

Scale bars 50 µm in **a**, **c, f**; 100 µm in **e, g** and **j**. Two-sided Mann-Whitney test was used for comparisons in **b, d, k and l**. Two-sided Fisher's exact test was used for comparisons in **n**. r, rostral; c, caudal; d, dorsal; v, ventral. "n" = number of animals used for the experiments. Source data are provided as a Source Data file.

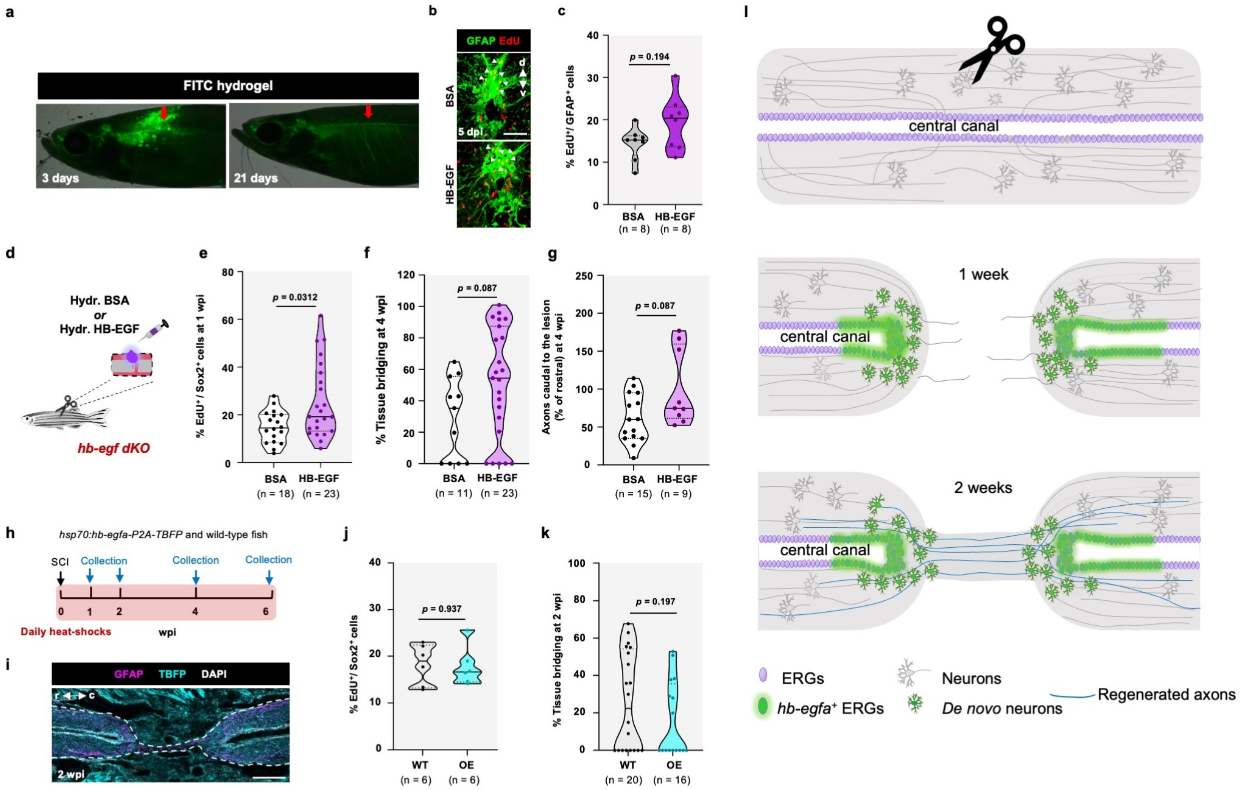

Supplementary Fig. 5. Hydrogel and transgene-mediated increases in Hb-egf levels.

(a) Administration of FITC-loaded hydrogels to sites of spinal cord injury. Live zebrafish underwent a single injection of FITC-loaded hydrogel adjacent to the usual transection site, and were imaged 2 and 21 days post injection. Red arrows indicate approximate injection site. One representative fish per group is shown.

(b, c) Cycling of GFAP-positive glial cells assessed by EdU (red) incorporation in spinal cords of fish treated with vehicle (BSA)- or HR-HB-EGF-loaded hydrogel. Analyses were performed at 5 days post injury (dpi). N = 2.

(d) Cartoon showing the strategy used for rescue experiements.

(e-g) Quantification of cycling ERGs, tissue bridging and axon crossing in *hb-egfa* dKO animals treated with HR-HB-EGF. N=3.

(h) Timeline for heat shock experiments and assessment of ependymal cell proliferation, bridging, axon growth and swim capacity in *hb-egfaOE* zebrafish. All animals, transgenics and controls underwent daily heat-shocks.

(i) Longitudinal section of *hb-egfa*OE spinal cord at 2 wpi showing TBFP expression, a proxy for Hb-egfa (cyan), along the rostrocaudal spinal cord axis. GFAP (magenta) stains glial cells. N = 2.

(j) Ependymal (Sox2^{+}) cell cycling assessed by EdU incorporation in wild-type and *hb-egfaOE* adult spinal cords at 1 wpi.

(k) Percentage of tissue bridging in wild-type and *hsp70:hb-egfOE* zebrafish at 2 wpi.

(l) Preliminary model summarizing Hb-egf effects during zebrafish spinal cord regeneration. Spinal cord injury triggers expression of *hb-egfa* in ERGs around the lesion site. Hb-egf acts on ERGs where it regulates their self-renewal and their differentiation

into neurons in and around the injury site, detectable as early as 1 wpi. Axon crossing and recovery of motor function occur subsequently.
Scale bar 50 μm in **b** and 200 μm in **i**. A two-sided Mann-Whitney test was used for comparisons in **c**, **e**, **f**, **g**, **j** and **k**. r, rostral; c, caudal; d, dorsal; v, ventral. n= number of animals used for the experiments. "n" = number of animals used for experiments. Source data are provided as a Source Data file.

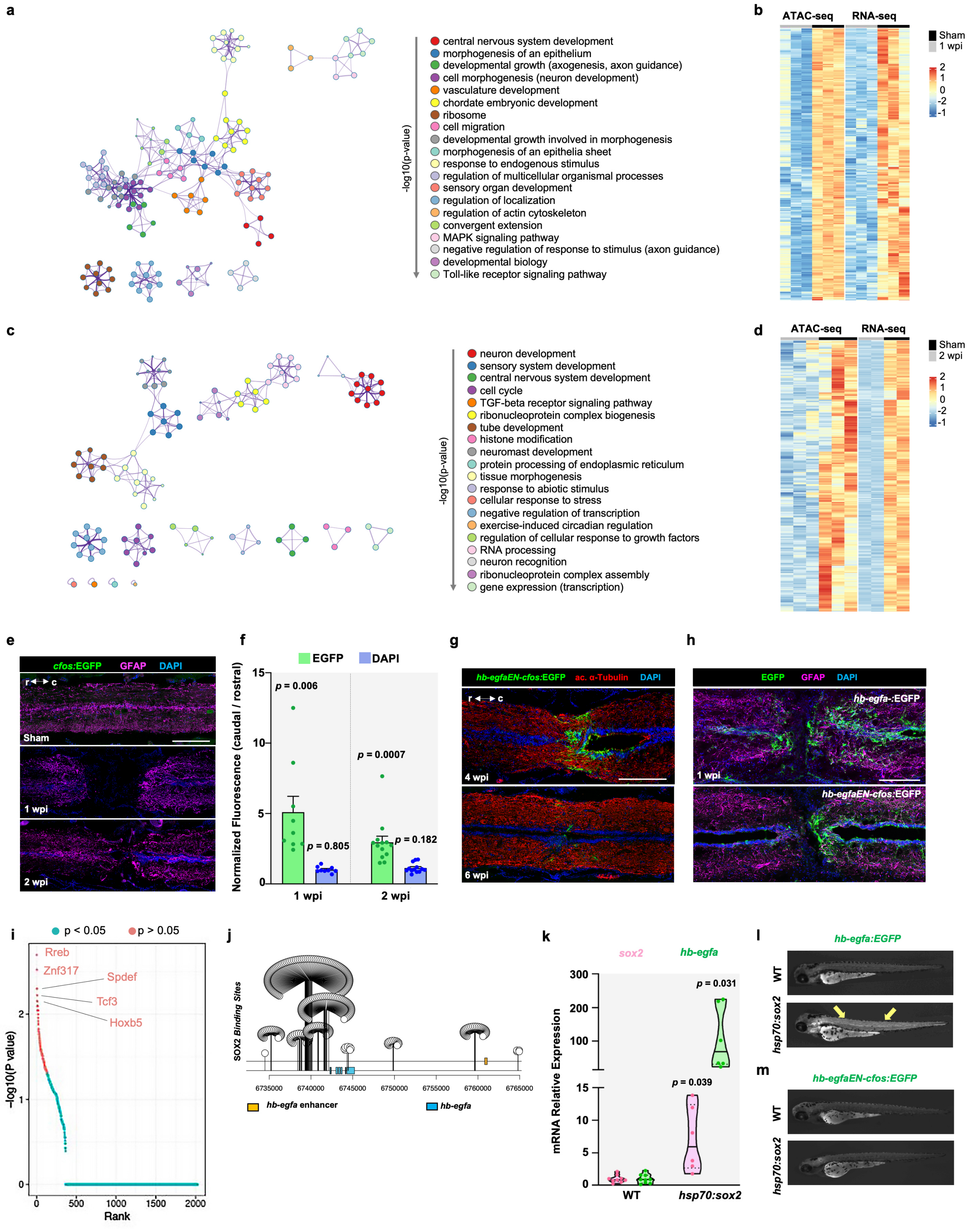
a
central nervous system development
morphogenesis of an epithelium
developmental growth (axogenesis, axon guidance)
cell morphogenesis (neuron development)
vasculature development
chordate embryonic development
ribosome
cell migration
developmental growth involved in morphogenesis
morphogenesis of an epithelia sheet
response to endogenous stimulus
regulation of multicellular organismal processes
sensory organ development
regulation of localization
regulation of actin cytoskeleton
convergent extension
MAPK signaling pathway
negative regulation of response to stimulus (axon guidance)
developmental biology
Toll-like receptor signaling pathway
-log10(p-value)
b
ATAC-seq
RNA-seq
Sham
1 wpi
c
neuron development
sensory system development
central nervous system development
cell cycle
TGF-beta receptor signaling pathway
ribonucleoprotein complex biogenesis
tube development
histone modification
neuromast development
protein processing of endoplasmic reticulum
tissue morphogenesis
response to abiotic stimulus
cellular response to stress
negative regulation of transcription
exercise-induced circadian regulation
regulation of cellular response to growth factors
RNA processing
neuron recognition
ribonucleoprotein complex assembly
gene expression (transcription)
-log10(p-value)
d
ATAC-seq
RNA-seq
Sham
2 wpi
e
cfos:EGFP GFAP DAPI
Sham
1 wpi
2 wpi
f
EGFP
DAPI
p = 0.006
p = 0.805
p = 0.0007
p = 0.182
Normalized Fluorescence (caudal / rostral)
1 wpi
2 wpi
g
hb-egfaEN-cfos:EGFP ac. α-Tubulin DAPI
4 wpi
6 wpi
h
EGFP GFAP DAPI
hb-egfa-:EGFP
1 wpi
hb-egfaEN-cfos:EGFP
i
p < 0.05
p > 0.05
Rreb
Znf317
Spdef
Tcf3
Hoxb5
-log10(P value)
Rank
j
SOX2 Binding Sites
hb-egfa enhancer
hb-egfa
k
sox2
hb-egfa
p = 0.031
p = 0.039
mRNA Relative Expression
WT
hsp70:sox2
l
hb-egfa:EGFP
WT
hsp70:sox2
m
hb-egfaEN-cfos:EGFP
WT
hsp70:sox2

Supplementary Fig. 6. Bioinformatic analyses of ATAC-seq and RNA-seq data and characterization of *hb-egfaEN*.

(a-d) Gene ontology of genes with associated chromatin regions after spinal cord injury at 1 **(a)** and 2 **(c)** weeks post injury (wpi) (also shown in **Supplementary Data 3** and **5)**. Heat maps show increased transcripts linked to nearby differentially accessible chromatin regions in 1 wpi **(b)** and 2 wpi **(d)** spinal cords versus sham-injured.

(e) Longitudinal sections of *cfos:EGFP* spinal cord, at 1 and 2 wpi.

(f) Quantification of EGFP and DAPI fluorescence rostral and caudal to the lesion at 1 and 2 wpi in *hb-egfaEN-cfos:EGFP* spinal cords. n=9 and 13 animals at 1 and 2 wpi, respectively, exhamined over 2 independent experiments. An unpaired two-tailed t-test was used for comparisons. Error bars indicate SEM.

(g) Longitudinal sections of *hb-egfaEN-cfos:EGFP* spinal cords at 4 and 6 wpi, indicating reduction of fluorescence by 6 wpi. N = 3.

(h) Longitudinal sections of *hb-egfaEN-cfos:EGFP* (bottom) and *hb-egfa:EGFP* (top) spinal cords at 1 wpi (crush), indicating similar EGFP expression pattern to those observed after transection injury. N = 3.

(i) BiFET analyses indicating enriched transcription factor motifs within *hb-egfaEN*. Red, $p<0.05$; Blue, $p>0.05$. Peaks are analyzed based on the hypergeometric distribution.

(j) Dandelion plot showing predicted Sox2 binding sites in or near *hb-egfa* promoter and enhancer. Motifs assessed with motifmatchr package, $p < 0.0005$.

(k) *sox2* and *hb-egfa* mRNA expression levels (pink and green, respectively) in wild-type (WT) or *hsp70:sox2* larvae 3 hours after heat shock. Expression is shown as fold change vs non-heat shocked larvae, whose *sox2* and *hb-egfa* mRNA levels are = 1 (dashed horizontal line). N = 2. Each point represents a single animal. Unpaired two-tailed t-test with Welch's correction used for comparisons.

(l) EGFP expression in 3 dpf *hb-egfa:*EGFP (top) or *hsp70:sox2*; *hb-egfa:*EGFP larvae 6 hours after heat shock. Trunk fluorescence (arrows) is detectable. N = 2. n = 7 (WT) and 6 (*hsp70:sox2*).

(m) EGFP expression in *hb-egfaEN-cfos:EGFP* (top) or *hsp70:sox2*; *hb-egfaEN-cfos:EGFP* larvae 6 hours after heat shock. No trunk fluorescence is detectable. N = 2. n = 12 (WT) and 10 (*hsp70:sox2*).

Scale bars 200 µm. r, rostral; c, caudal.

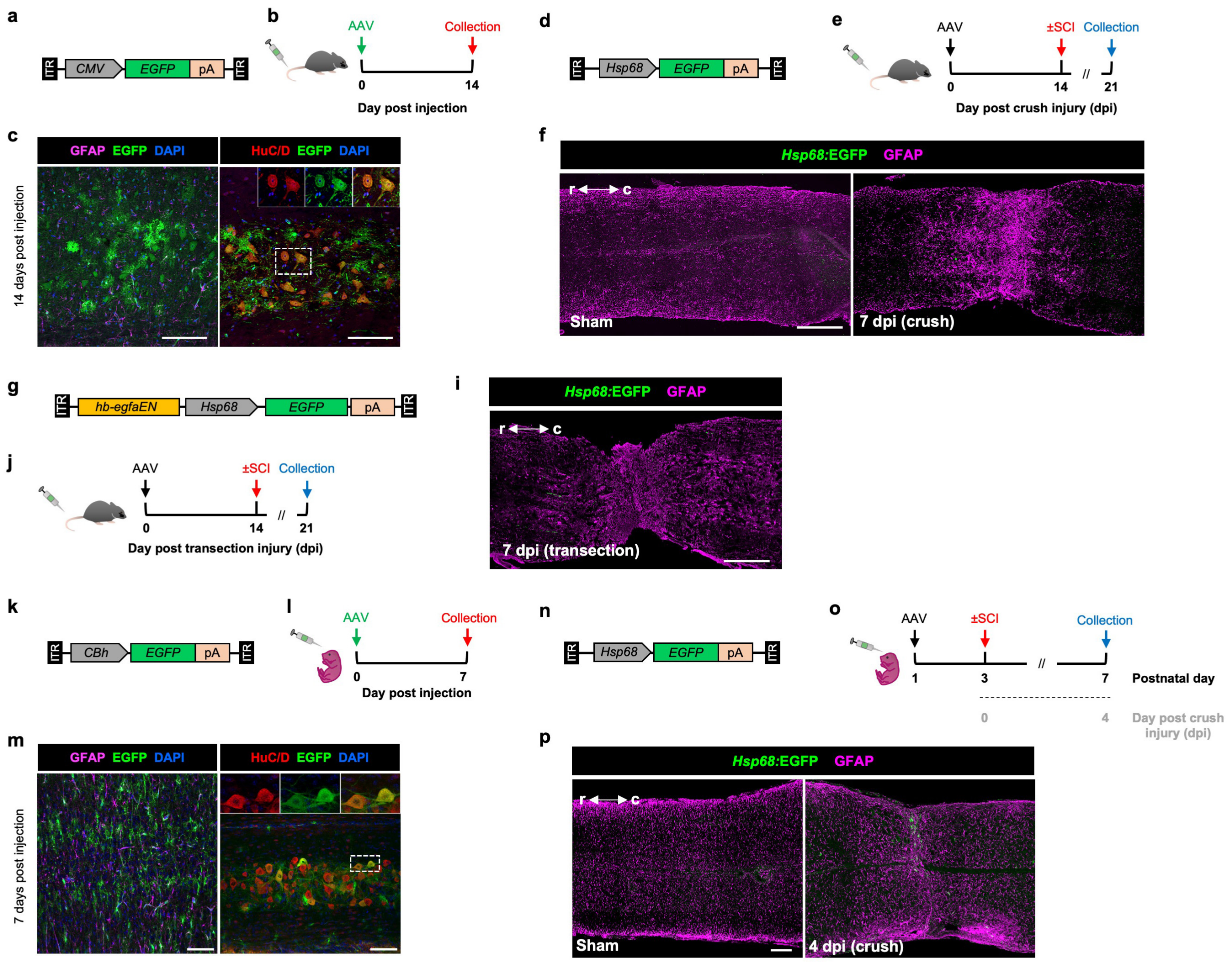

Supplementary Fig. 7. AAV-cc.47-mediated transduction of spinal cord cell types in neonatal and adult mice and characterization of control enhancer constructs.

(a, b) AAV construct **(a)** and experimental design **(b)** to test AAV-cc.47 transduction in adult spinal cord.

(c) Immunofluorescence staining of sections of uninjured adult spinal cord showing expression of EGFP, the glial marker GFAP, and the neuronal marker HuC/D at 14 days post transduction.

(d, e) AAV construct **(d)** and experimental design **(e)** for tests of *Hsp68:*EGFP expression in adult spinal cord after crush injury.

(f) Longitudinal sections of spinal cords from adult mice after sham-injury or 7 days post crush injury (dpi). Mice transduced with AAV-*Hsp68:EGFP* show little or no detectable EGFP at the site of injury.

(g, h) AAV construct **(g)** and experimental design **(h)** for tests of *hb-egfaEN-Hsp68:*EGFP expression in adult spinal cord after a transection injury.

(i) Immunofluorescence staining of sections of uninjured adult spinal cord showing expression of EGFP and the glial marker GFAP at 7 days post a transection injury.

(k, l) AAV construct **(k)** and experimental design **(l)** to test AAV transduction in neonatal spinal cord.

(m) Immunofluorescence staining of sections of uninjured neonatal spinal cord showing expression of EGFP, the glial marker GFAP, and the neuronal marker HuC/D at 7 days post injection.
(n, o) AAV construct **(n)** and experimental design **(o)** for tests of *Hsp68:*EGFP expression in neonatal spinal cord.
(p) Longitudinal sections of spinal cords from neonatal mice after sham injury or 4 dpi. Mice transduced with AAV-*Hsp68:EGFP* show little or no detectable EGFP at the site of injury.
Scale bars 100 μm in **c**, **m** and **p**; 500 μm in **f** and **i**. Dashed regions in **c** and **m** indicate magnified areas. r, rostral; c, caudal.

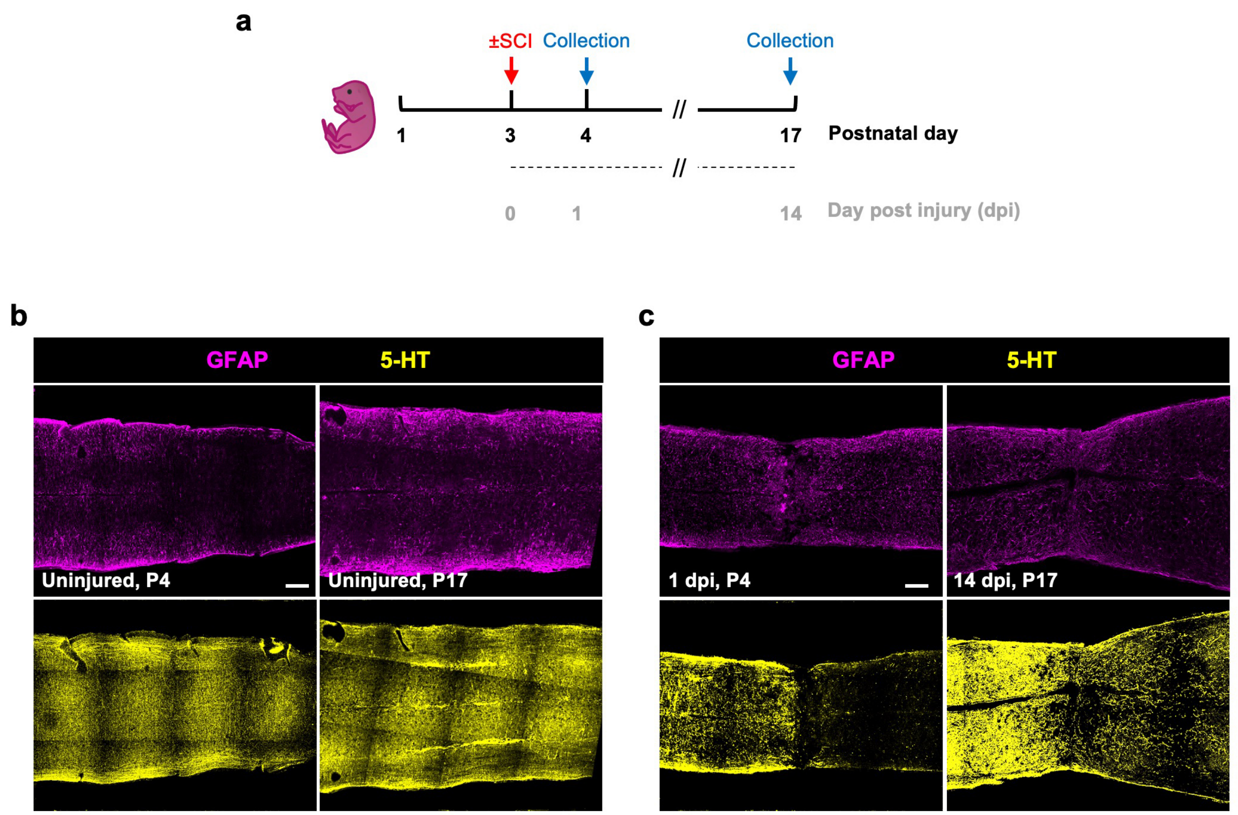

Supplementary Fig. 8. Stains of serotonergic neurons after spinal cord crush injury in neonatal mice.

(a) Experimental design.

(b) Longitudinal sections of spinal cords of uninjured mice collected at postnatal day 4 (P4) or postnatal day 17 (P17), stained with the glial marker GFAP and 5-HT, marking serotonergic axons of serotonergic neurons.

(c) Longitudinal sections of spinal cords after crush injury at P3, collected at P4 or P17, and stained with GFAP and 5-HT. 5-HT-positive axons (yellow) are visible caudal to the injury site at 14 dpi.

Scale bars 200 µm.

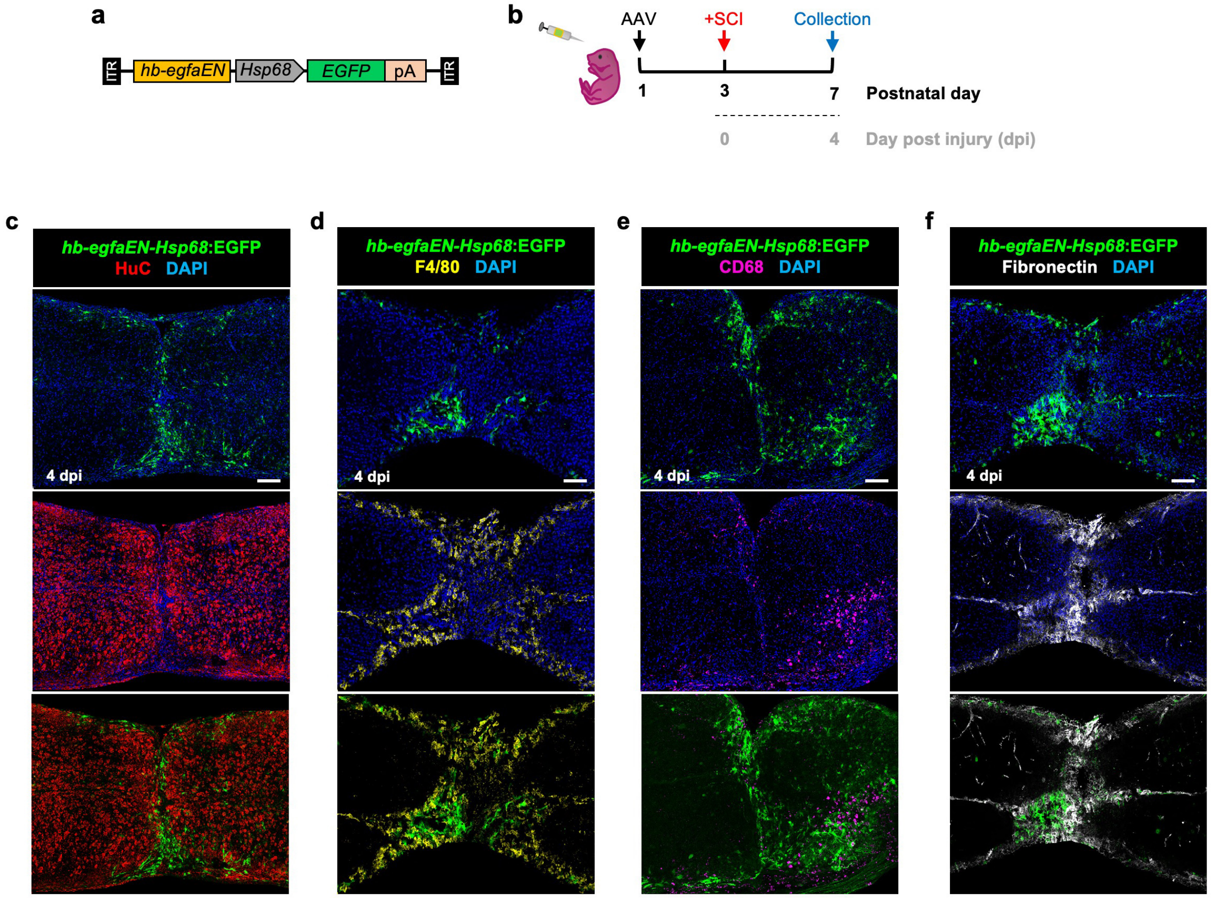

Supplementary Fig. 9. Characterization of cells expressing *hb-egfaEN-Hsp68:*EGFP after spinal cord injury in neonatal mice.
(a, b) Viral construct and experimental design.
(c) Expression of *hb-egfaEN*-directed EGFP and the neuronal marker HuC/D in spinal cord at 4 dpi.
(d) Expression of *hb-egfaEN*-directed EGFP and the macrophage marker F40/80 in neonatal spinal cord at 4 dpi.
(e) Expression of *hb-egfaEN*-directed EGFP and the microglial marker CD68 in spinal cord at 4 dpi.
(f) Expression of *hb-egfaEN*-directed EGFP and bridge-forming fibronectin in spinal cord at 4 dpi. Scale bars 100 μm.

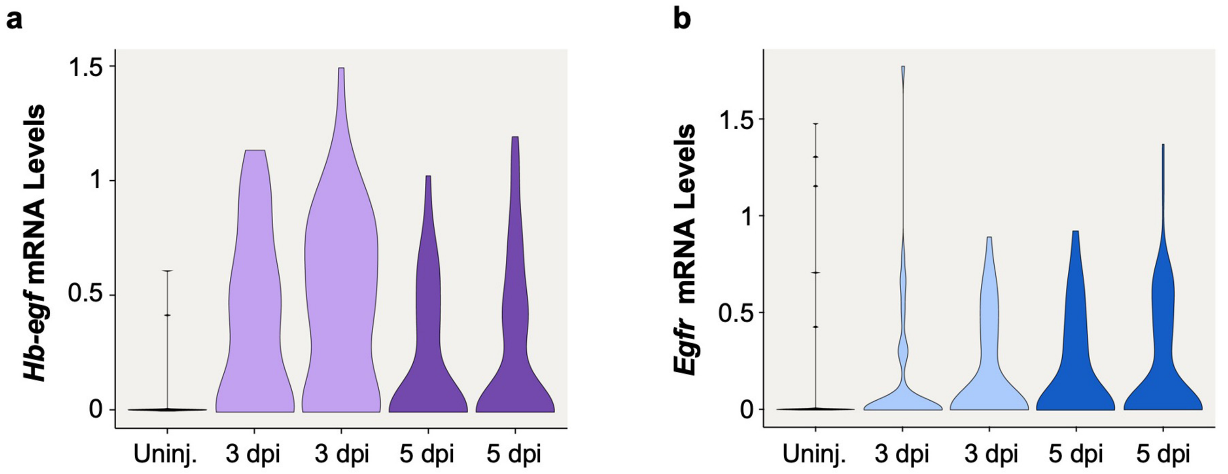

Supplementary Fig. 10. Expression of *Hb-egf* in neonatal mouse spinal cord.
(a) *Hb-egf* and **(b)** *Egfr* mRNA levels in astrocytes of uninjured neonatal mouse spinal cord, or 3 and 5 days after crush injury, based on published datasets (Li et al., 2020). Light purple in **a** and light blue in **b** are 2 different replicates at 3 dpi. Dark purple in **a** and dark blue in **b** are two different replicates at 5 dpi.
