# Supplementary material for: Spinal cord repair is modulated by the neurogenic factor Hb-egf under direction of a regeneration-associated enhancer
Source: Nat Commun. 2023 Aug 11;14:4857. doi: 10.1038/s41467-023-40486-5 (PMC10421883; doi:10.1038/s41467-023-40486-5)
Supplement: Supplementary file 3 — Description of Additional Supplementary Files [file 41467_2023_40486_MOESM3_ESM.pdf]

Description of Additional Supplementary Files

File Name: Supplementary Data 1
Description: Spinal cord RNA-seq profiling at 1 week post injury (wpi).

File Name: Supplementary Data 2
Description: Merged ATAC-seq peaks and RNA-seq at 1 wpi.

File Name: Supplementary Data 3
Description: Top 20 GO analyses terms of ATAC-peaks assigned to differentially expressed genes at 1 wpi.

File Name: Supplementary Data 4
Description: Merged ATAC-seq peaks and RNA-seq at 2 wpi.

File Name: Supplementary Data 5
Description: Top 20 GO analyses terms of ATAC-peaks assigned to differentially expressed genes at 2 wpi.

File Name: Supplementary Movie 1
Description: Video of wild-type fish in an isolated breeder tank at 4 wpi. As reflected in the swim capacity violin plots in Fig. 2, there is variability in motor recovery between animals, with ~70-80% of the wild-type fish able to swim against increasing water current at 4 wpi.

File Name: Supplementary Movie 2
Description: Video of *hb-egf* dKO fish in an isolated breeder tank at 4 wpi. Many of the animals are unable to remain upright and swim, indicated continued partial or full paralysis distal to the injury.

File Name: Supplementary Movie 3
Description: Video of *hb-egfb*KO fish in an isolated breeder tank at 4 wpi. Most animals recover full swim capacity, similar to wild-types.

File Name: Supplementary Movie 4
Description: Video of *hb-egfa*KO fish in an isolated breeder tank at 4 wpi. Many or most animals have not recovered full swim capacity.

File Name: Supplementary Movie 5
Description: Video of wild-type fish in an isolated breeder tank at 2 wpi and daily heat-shocks. As reflected in the swim violin plots in Fig. 3, there is variability in recovery between animals, with ~50% of the control fish able to swim against increasing water current at 2 wpi.

File Name: Supplementary Movie 6
Description: Video of *hsp70:hb-egfa-P2A-TBFP* clutchmates at 2 wpi and daily heat-shocks. Many or most animals have not recovered full swim capacity.

File Name: Supplementary Movie 7
Description: Video showing motor function of a neonatal mouse at 30 dpi. Mice received AAV carrying an *hb-egfaEN-hsp68:EGFP* construct at P1 and underwent spinal cord crush injury at P3.

File Name: Supplementary Movie 8
Description: Video showing motor function of a neonatal mouse at 30 dpi. Mice received AAV carrying an *hb-egfaEN-hsp68:HB-EGF* construct at P1 and underwent spinal cord crush injury at P3.
