# Supplementary material for: Spinal cord repair is modulated by the neurogenic factor Hb-egf under direction of a regeneration-associated enhancer
Source: Nat Commun. 2023 Aug 11;14:4857. doi: 10.1038/s41467-023-40486-5 (PMC10421883; doi:10.1038/s41467-023-40486-5)
Supplement: Supplementary file 17 — Reporting Summary [file 41467_2023_40486_MOESM17_ESM.pdf]

nature portfolio

Corresponding author(s): Kenneth D. Poss

Last updated by author(s): Jul 16, 2023

Reporting Summary

Nature Portfolio wishes to improve the reproducibility of the work that we publish. This form provides structure for consistency and transparency in reporting. For further information on Nature Portfolio policies, see our Editorial Policies and the Editorial Policy Checklist.

Please do not complete any field with "not applicable" or n/a. Refer to the help text for what text to use if an item is not relevant to your study.
For final submission: please carefully check your responses for accuracy; you will not be able to make changes later.

Statistics

For all statistical analyses, confirm that the following items are present in the figure legend, table legend, main text, or Methods section.

n/a	Confirmed	
☐	☒	The exact sample size (*n*) for each experimental group/condition, given as a discrete number and unit of measurement
☐	☒	A statement on whether measurements were taken from distinct samples or whether the same sample was measured repeatedly
☐	☒	The statistical test(s) used AND whether they are one- or two-sided<br>*Only common tests should be described solely by name; describe more complex techniques in the Methods section.*
☒	☐	A description of all covariates tested
☐	☒	A description of any assumptions or corrections, such as tests of normality and adjustment for multiple comparisons
☐	☒	A full description of the statistical parameters including central tendency (e.g. means) or other basic estimates (e.g. regression coefficient) AND variation (e.g. standard deviation) or associated estimates of uncertainty (e.g. confidence intervals)
☐	☒	For null hypothesis testing, the test statistic (e.g. *F*, *t*, *r*) with confidence intervals, effect sizes, degrees of freedom and *P* value nc<br>*Give P values as exact values whenever suitable.*
☒	☐	For Bayesian analysis, information on the choice of priors and Markov chain Monte Carlo settings
☒	☐	For hierarchical and complex designs, identification of the appropriate level for tests and full reporting of outcomes
☒	☐	Estimates of effect sizes (e.g. Cohen's *d*, Pearson's *r*), indicating how they were calculated

Our web collection on statistics for biologists contains articles on many of the points above.

Software and code

Policy information about availability of computer code

Data collection	All confocal images were acquired with Zeiss LSM700 confocal microscope with Zen 2010 B SP1.<br>In situ hybridization images were acquired with a Leica6000 compound microscope with Leica Application Suite X (v.3.4.2).<br>Dissecting scope images were acquired with a Zeiss Axio Zoom microscope with Zen pro 2012.<br>qPCR was performed using a Roche light cycler 480 with software 1.5.0 SP4.<br>Single cell sequencing was performed using 10X Genomics instrument and pipeline.<br>Swim capacity was measured using a swim tunnel respirometer device (Loligo, SW100605L, 120V/60Hz)
Data analysis	All graphs and statistical analyses were generated using Prism v9.<br>Measurement of tissue bridge and biocytin labeling was performed using Fiji (v.2.3.0)<br>Software used for bioinformatic include the following: Metascape (v3.5.20230501), Panther (v17.0), Ingenuity Pathway Analysis (IPA, IPA, v. 70750971), Seurat package (v 4.1.1), CellChat (v 1.4.0), R scripts 'ScType' (v 1.0), Bowtie2 v 2.2.5, samtools (v 1.3.1), MACS2 (v 2.1.0), csaw (v 1.20.0), DiffBind (v 2.14.0), Tophat2 (v2.1.1), htseq-count (v0.6.0), DESeq2 (v1.26.0), ChIPpeakAnno (v3.20.1), circus (v0.69-8), JASPAR (v2018), trackViewer (v 1.22.1), ComplexHeatmap (v 2.2.0).

For manuscripts utilizing custom algorithms or software that are central to the research but not yet described in published literature, software must be made available to editors and reviewers. We strongly encourage code deposition in a community repository (e.g. GitHub). See the Nature Portfolio guidelines for submitting code & software for further information.

Data

Policy information about availability of data

All manuscripts must include a data availability statement. This statement should provide the following information, where applicable:

- Accession codes, unique identifiers, or web links for publicly available datasets
- A description of any restrictions on data availability
- For clinical datasets or third party data, please ensure that the statement adheres to our policy

The bulk RNA, ATAC and single cell RNA sequencing datasets generated in this study have been deposited at at GEO. The accession number for bulk RNA and ATAC sequencing is GSE193503. The accession number for single cell RNA-seq data is GSE213435. All reagents, data and zebrafish lines generated in this study are available from the corresponding author upon request. Source Data are provided with this manuscript.

Human research participants

Policy information about studies involving human research participants and Sex and Gender in Research.

Reporting on sex and gender	Not applicable
Population characteristics	Not applicable
Recruitment	Not applicable
Ethics oversight	Not applicable

Note that full information on the approval of the study protocol must also be provided in the manuscript.

Field-specific reporting

Please select the one below that is the best fit for your research. If you are not sure, read the appropriate sections before making your sel[illegible]n.

☒ Life sciences ☐ Behavioural & social sciences ☐ Ecological, evolutionary & environmental sciences

For a reference copy of the document with all sections, see nature.com/documents/nr-reporting-summary-flat.pdf

Life sciences study design

All studies must disclose on these points even when the disclosure is negative.

Sample size	No statistical methods were used to pre-determine sample sizes. Number of animals in each group was determined based on previous studies (Mokalled et al., 2016) and taking into account the considerable variability in assays of spinal cord regeneration capacity. All data are provided as violin plots with each data point indicated.
Data exclusions	All animals analysed were included in the data.
Replication	All experiements were repeated 2-4 times, as indicated in the figure legends.
Randomization	Male and female animals were included in the experiments, and randomly assigned to control or experimental group.
Blinding	Researchers were blinded for swim assay experiments. For experiments employing mutant/transgenic fish or requiring repeated H R-HB-EGF and BSA treatment, experiments were performed in a stepwise manner thus investigators were not blinded. For experiments with neonatal mice, each litter was treated either with control or experimental viruses, then placed back in the mother's cage and analyzed in an unblinded manner.

Reporting for specific materials, systems and methods

We require information from authors about some types of materials, experimental systems and methods used in many studies. Here, indicate whether each material, system or method listed is relevant to your study. If you are not sure if a list item applies to your research, read the appropriate section before selecting a response.

Materials & experimental systems

n/a Involved in the study

- [ ] [x] Antibodies
- [ ] [x] Eukaryotic cell lines
- [x] [ ] Palaeontology and archaeology
- [ ] [x] Animals and other organisms
- [x] [ ] Clinical data
- [x] [ ] Dual use research of concern

Methods

n/a Involved in the study

- [x] [ ] ChIP-seq
- [x] [ ] Flow cytometry
- [x] [ ] MRI-based neuroimaging

Antibodies

Antibodies used

- Primary antibodies:
Rabbit anti-GFP (Life Technologies, A11122, 1:200)
Chicken anti-GFP (Aves Labs, GFP-1020, 1:500)
Mouse anti-GFAP (ZIRC, Zrf1, 1:1000)
Rabbit anti-GFAP (Sigma, G9269, 1:200)
Mouse anti-GFAP (Sigma, G3893, 1:500)
Rabbit anti-Sox2 (Abcam, ab97959, 1:200)
Mouse anti-HuC/D (Invitrogen, A-21271, 1:100)
Mouse anti-acetylated-α-tubulin (Sigma, T6793, 1:1000)
Rabbit anti-dsRed (Clontech, 632496, 1:200)
Goat anti-5-HT (Immunostar, 20079 1:5,000)
Rabbit anti-fibronectin (Sigma, F3648, 1:200)
Rat anti-CD68 (Bio-Rad, MCA1957, 1:600)
Rat anti F4/80 (Biorad, MCA497R)
Rabbit anti-Ki67 (Abcam, ab15580, 1:200)

- Secondary antibodies:
Goat anti-Mouse IgG (H+L) Highly Cross-Adsorbed Secondary Antibody, Alexa Fluor 488 (Thermo Scientific, A11029, 1:200 dilution).
Goat anti-Mouse IgG (H+L) Highly Cross-Adsorbed Secondary Antibody, Alexa Fluor 594 (Thermo Scientific, A11032, 1:200 dilution).
Goat anti-Mouse IgG (H+L) Highly Cross-Adsorbed Secondary Antibody, Alexa Fluor 633 (Thermo Scientific, A21052, 1:200 dilution).
Goat anti-Rabbit IgG (H+L) Highly Cross-Adsorbed Secondary Antibody, Alexa Fluor 594 (Thermo Scientific, A11037, 1:200 dilution).
Goat anti-Rabbit IgG (H+L) Highly Cross-Adsorbed Secondary Antibody, Alexa Fluor 488 (Thermo Scientific, A11034, 1:200 dilution).
Goat anti-Rabbit IgG (H+L) Highly Cross-Adsorbed Secondary Antibody, Alexa Fluor 633 (Thermo Scientific, A21071, 1:200 dilution).
Goat anti-Chicken IgG (H+L) Highly Cross-Adsorbed Secondary Antibody, Alexa Fluor 488 (Thermo Scientific, A11039, 1:200 dilution).
Goat anti-Rat IgG (H+L) Highly Cross-Adsorbed Secondary Antibody, Alexa Fluor 568 (Thermo Scientific, A11007, 1:200 dilution).
Rabbit anti-Goat IgG (H+L) Highly Cross-Adsorbed Secondary Antibody, Alexa Fluor 594 (Thermo Scientific, A11080, 1:200 dilution).
Donkey anti-Goat IgG (H+L) Highly Cross-Adsorbed Secondary Antibody, Alexa Fluor 488 (Thermo Fisher Scientific, A11055, 1:200 dilution)
Donkey anti-Goat IgG (H+L) Highly Cross-Adsorbed Secondary Antibody, Alexa Fluor 594 (Thermo Fisher Scientific, A11058, 1:200 dilution)

Validation

All antibodies used in this study are commercially available, and were validated by the respective commercial source in either human, murine, or zebrafish tissue for application in IF, IHC-P or WB.
Primary antibodies have previously employed in the following studies:
Rabbit anti-GFP (Life Technologies, A11122): Shoffner et al., 2021; Yan et al., 2023; Goldman et al., 2017
Chicken anti-GFP (Aves Labs, GFP-1020): Yan et al., 2023
Mouse anti-GFAP (ZIRC, Zrf-1, 1:200): Hevia et al., 2022
Rabbit anti-GFAP (Sigma, G9269, 1:200): Saadoun et al., 2022
Mouse anti-GFAP (Sigma, G3893, 1:500): Min-Hui et al., 2019
Rabbit anti-Sox2 (Abcam, ab97959, 1:200): Shi et al., 2023
Mouse anti-HuC/D (Invitrogen, A-21271, 1:100): Babu et al., 2022
Mouse anti-acetylated-α-tubulin (Sigma, T6793, 1:1000): Mokalled et al., 2016
Rabbit anti-dsRed (Clontech, 632496, 1:200): Shoffner et al., 2021
Goat anti-5-HT (Immunostar, 20079, 1:5,000): Li et al., 2020
Rabbit anti-fibronectin (Sigma, F3648, 1:200): Zheng et al., 2023
Rat anti-CD68 (Bio-Rad, MCA1957, 1:600): Qian et al., 2022
Rat anti F4/80 (Biorad, MCA497R, 1:200): Riera-Domingo et al., 2023
Rabbit anti-Ki67 (Abcam, ab15580, 1:200): Yan et al., 2023

Secondary antibodies have been employed in Shoffner et al., 2021; Yan et al., 2023; Han et al., 2018; Goldman et al., 2017, Thaddeus et al., 2023; Espinosa-Jiménez et al., 2023, among many others.

Eukaryotic cell lines

Policy information about cell lines and Sex and Gender in Research

Cell line source(s)

We used HEK293s for AAV packaging. HEK293s were obtained from the University of North Carolina Vector Core.

Authentication	The cell lines were not authenticated.
Mycoplasma contamination	The cell lines tested negative for mycoplasma.
Commonly misidentified lines (See ICLAC register)	This study does not involve commonly misidentified cell lines.

Animals and other research organisms

Policy information about studies involving animals; ARRIVE guidelines recommended for reporting animal research, and Sex and Gender in Research

Laboratory animals	This study involved wild-type and transgenic fish of the outbred Ekkwill strain. Zebrafish had an age range from 6-12 months old and measured ~2.5 cm in length at the time of spinal cord injury. The list of transgenic lines used in the manuscript is reported in Methods.<br>Neonatal C57BL6 mice received viral injections at postnatal day 1 and underwent spinal cord crush injury at postnatal day 3. Adult C57BL6 mice were injected with viruses at 3 months old and underwent spinal cord injury one week after viral injection. Mice were housed in a temperature-controlled (~18-23C, 40–60% humidity) and enriched environment, with a 12h light/dark cycle, and provided standard chow and water.
Wild animals	This study does not involve wild animals.
Reporting on sex	Both male and female animals were used for the experiments. No differences in regeneration capacity have been reported between the two sexes.
Field-collected samples	No field collected samples were used for this study.
Ethics oversight	Animal use was approved by the Institutional Animal Care and Use Committee at Duke University, Protocol #A005-21-0l for zebrafish and Protocol #A003-22-01 for mice.

Note that full information on the approval of the study protocol must also be provided in the manuscript.

This checklist template is licensed under a Creative Commons Attribution 4.0 International License, which permits use, sharing, adaptation, distribution and reproduction in any medium or format, as long as you give appropriate credit to the original author(s) and the source, provide a link to the Creative Commons license, and indicate if changes were made. The images or other third party material in this article are included in the article's Creative Commons license, unless indicated otherwise in a credit line to the material. If material is not included in the article's Creative Commons license and your intended use is not permitted by statutory regulation or exceeds the permitted use, you will need to obtain permission directly from the copyright holder. To view a copy of this license, visit http://creativecommons.org/licenses/by/4.0/
